# The involvement of exosomes in the tumorigenicity of breast cancer cell lines through the crosstalk between STAT3, Notch, and Wnt signaling pathways

**DOI:** 10.1007/s12672-025-03334-0

**Published:** 2025-09-15

**Authors:** Shymaa Abdullah Mohamed, Toka M. I. Badawy, Menna Allah M. K. Hussein, Mohammed Salama, Yasser M. El Kerm, Mohamed A. Abdel Mohsen

**Affiliations:** 1 Molecular Biology Unit, Medical Technology Center, Applied Medical Chemistry Department, Medical Research Institute, Alexandria University, Alexandria, Egypt; 2 Applied Medical Chemistry Department, Medical Research Institute, M.Sc, Alexandria University, Alexandria, Egypt; 3 Histochemistry and Cell Biology Department, Medical Research Institute, Alexandria University, Alexandria, Egypt; 4 Cancer Management and Research Department, Medical Research Institute, Alexandria University, Alexandria, Egypt

**Keywords:** Triple negative breast cancer, Exosomes, Tumorigenic behavior, STAT3 signaling pathway, Wnt signaling pathway, Notch signaling pathway

## Abstract

**Background:**

Exosomes play a critical role in the tumor microenvironment by interacting with signaling pathways that facilitate breast cancer metastasis, particularly the STAT3 pathway. The STAT3 pathway is essential for tumor progression and aggressiveness, as it interacts with other pathways like Wnt and Notch in particular, triple-negative breast cancer (TNBC) which is the most aggressive form of breast cancer, with a poor prognosis and rapid metastasis. It is exciting to researchers because it is therapeutically challenging and highly invasive. As a result of the lack of specific treatment options, conventional therapy is widely used, which frequently results in relapse.

**Objectives:**

Elucidate the critical roles of exosomes in modulating breast cancer behaviour and disease progression in TNBC through STAT3 signaling pathways. Specifically, it was focused on the interplay between STAT3 and Wnt or Notch signaling

**Materials and methods:**

Exosomes were isolated from one TNBC patient and characterized using electron microscopy and the western blotting technique. This study utilized two subtypes of breast cancer cell lines: non-TNBC and TNBC. AG490 treatment inhibited STAT3 signaling, and then after inhibition, tumorigenic behaviours were evaluated. Gene expression profiles related to the investigated signaling pathways, Wnt and Notch, were detected.

**The results and conclusion:**

Exosomes significantly affect tumor behaviours and chemoresistance and manipulate signaling pathways associated with tumorigenesis, including Wnt/β-catenin and Notch. These results demonstrated the tumorigenic role of exosomes in the TNBC aggressiveness and suggest that their mechanisms may involve Wnt or Notch signaling mediated by the STAT3.

**Graphical abstract:**

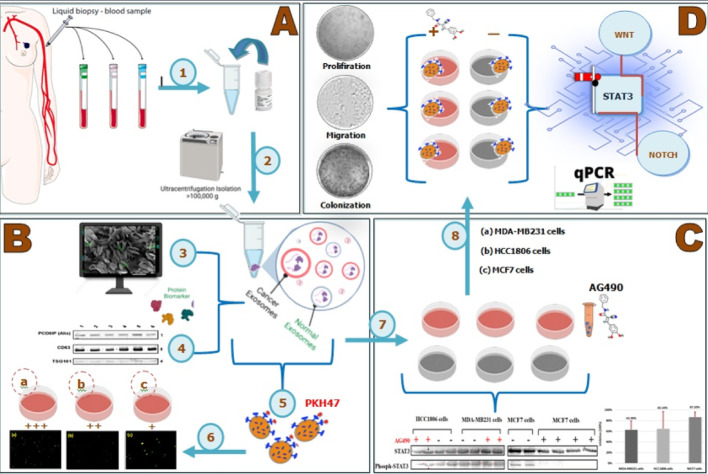

**Supplementary Information:**

The online version contains supplementary material available at 10.1007/s12672-025-03334-0.

## Introduction

**Fig. 1:**
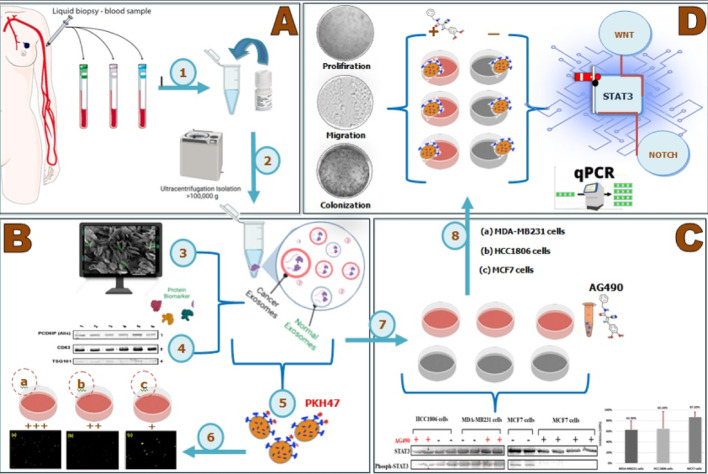
Graphical Abstract. **A** 1. Exosomes isolation from serum, **2.** Purification with ultracentrifuge. **B** Exosomes characterization, **3.** Scanning electron microscope and **4.** Western blotting then **5.** Labeling with PKH47 dye followed by assessment of **6.** Uptake efficiency by three cell lines **C 7.** STAT3 pathway inhibition using AG490. **D 8.** Crosstalk assessment between STAT3, WNT and NOTCH signalling

Breast cancer is a disease with a wide range of histology, cellular origins, mutations, metastatic potential, disease progression, therapeutic response, and clinical outcomes [1]. Each provides signatures that categorize breast cancers as luminal A (proximal), luminal B (distal), luminal human epidermal growth factor-2 (HER-2), or basal-like phenotypes [2]. Triple-negative breast cancer (TNBC) is a type of aggressive breast cancer that is renowned for a lack of expression of both the estrogen and the progesterone receptors, as well as the absence of HER2 overexpression. TNBC tumors have relatively simple molecular phenotypes but are inherently heterogeneous and exhibit diverse morphology, signaling pathway activity, and gene expression. Thus, they have complex clinicopathological features that adversely affect the prognosis of TNBC patients. TNBC has recently been categorized into six subtypes with distinct gene expression patterns: basal-like 1 (BL1), basal-like 2 (BL2), mesenchymal stem-like (MSL), luminal androgen receptor (LAR), immunomodulatory (IM), and mesenchymal (M). Lacking TNBC target receptors complicates treatment and predisposes patients to rapid metastasis, treatment resistance, and recurrence [3]. TNBC can withstand hormone and immunotherapy but is often sensitive to chemotherapeutic agents and radiotherapy [2]. Although early TNBC may respond to standard chemotherapy, traditional hormone therapies, and targeted agents are ineffective in this cancer type [4]. Doxorubicin (DOXO) is considered the most effective chemotherapeutic medication for breast cancer; however, the effectiveness of DOXO is negatively affected by multidrug resistance during chemotherapy [5]. Therefore, a better understanding of the tumor microenvironment (TME) and molecular mechanisms underlying TNBC may aid in the identification of therapeutic targets as well as predictive or prognostic biomarkers, as well as an understanding of the signaling pathways of response or failure to current cancer treatments (Fig. 1).

Exosomes are nanoscale membrane vesicles secreted by nearly all eukaryotic cells [5] characterized by their heterogeneity in size, content, functional impact on recipient cells, and cellular origin [6]. Exosome heterogeneity is influenced by their sources, as those derived from various tissues or organs exhibit distinct biological activities, including cancer cell-derived exosomes [7]. In the 1980s, Eberhard Trams and Rose Johnstone designated extracellular vesicles (EVs) as “exosomes,” with size serving as a primary distinguishing characteristic at the time [6]. EVs are segregated into three types based on their size and biogenesis: exosomes (30–200 nm), which have an endocytic origin; macrovesicles (100–1000 nm), which are produced by budding and blubbing from the plasma membrane; and apoptotic bodies (> 1000 nm), are released by cells undergoing programmed cell death and signal cell engulfment. Structurally, exosomes are lipid bilayer membrane vesicles with embedded transmembrane tetraspanin proteins, including the common differentiation markers (CD9, CD63, and CD81; ALG-2-interacting protein X (Alix) and tumor susceptibility gene 101 (TSG101) [7–10]. These markers can help identify and classify exosomes. Functionally, They facilitate cell-to-cell communication by engulfing functional biomolecules such as proteins, RNA, DNA, and lipids [11, 12]. In addition, exosomes, as messenger carriers, transport various active biomolecules from host cells to recipient cells because they are cell-derived membranous structures capable of easily binding to and releasing their contents into another cell via membrane fusion [13].

Exosomes, a part of the TME, help tumors grow and spread. They facilitate communication between cells within the TME, acting like a signal bridge that triggers angiogenesis, migration, adhesion, and invasiveness. The complex interplay between exosomes and signaling pathways makes breast TME diverse and complicated, which contributes to the progression and metastasis of breast cancer [14]. The signal transducer and activator of transcription 3 (STAT3) signaling pathway plays a critical role in TNBC immune evasion [15]. The oncogenic potential of STAT3 is widely recognized through its role in regulating the expression of genes associated with cancer cell tumorigenic behavior, chemoresistance, immune suppression, stem cell self-renewal, and maintenance [16, 17]; STAT3 is overexpressed and constitutively activated and strongly associated with tumor initiation, progression, metastasis, chemotherapy resistance, and poor survival rates [18]. Moreover, crosstalk between STAT3 and the Wnt or Notch signaling exhibits different effects on typical processes that regulate cell fate [19–21]. The wingless-related integration site (Wnt) signaling pathway has recently been identified as a cause of a variety of human pathologies, including breast cancer, and was active in prehistoric and evolutionary times, controlling critical aspects of cell fate determination, migration, and polarity [18, 22]. The Neurogenic locus notch homolog protein (Notch) signaling pathway plays a role in the pathogenesis and progression of TNBC. Firstly, Notch receptors link to the behavior of tumor-initiating cells and the development of TNBCs. Secondly, the Notch pathway plays a significant role in mammary cancer stem cell maintenance and expansion. Finally, Notch receptor expression and activation are strongly associated with the aggressive clinicopathological and biological phenotypes of breast cancer (such as invasiveness and chemoresistance), that are crucial characteristics of the TNBC subtype [23].

Therefore, the main objective is the study of crucial roles of exosomes in breast cancer behaviour, drug resistance, and progression *via* STAT3 signaling pathways in TNBC. In particular, it focuses on the crosstalk between STAT3 and either Wnt or Notch signaling.

## Materials and methods

### Cell lines

The breast cancer cell lines MDA-MB-231, mesenchymal-like; HTB-26™, HCC1806, basal-like; CRL-2335™, and MCF7, estrogen receptor-positive; CRL3435™, were purchased from ATCC (Manassas, Virginia, USA). MDA-MB-231, MCF7, and HCC1806 cells were cultured in high-glucose Dulbecco’s Modified Eagle Medium (DMEM), low-glucose DMEM, and RPMI1640 media, respectively. All media were supplemented with 10% fetal bovine serum (FBS) and protected with 0.1 mg/ml penicillin/streptomycin solution.

### Exosomes isolation

A serum sample was isolated from a TNBC woman with stage IV and grade III, and the tumor size was 8*7 cm. Informed consent was obtained in writing to the patient in compliance with the Ethics Committee of the Medical Research Institute at Alexandria University (E/C.S/N.T91/2021). Exosomes were isolated from serum using an exosome isolation kit (Invitrogen™ UAB). For purification, the pellet was diluted with 7.5 ml of phosphate-buffered saline (PBS), mixed well, and ultracentrifuged at 100,000 ×g for 90 min. This step was repeated twice and then resuspended in 400 µl of PBS.

### Size characterization of exosomes

The size of exosomes was defined using scanning and transmission electron microscope (SEM & TEM). The exosomal pellet was resuspended in 1 ml of Dulbecco’s phosphate-buffered saline (DPBS) and fixed in a 2% paraformaldehyde solution. After fixation, the samples were diluted with distilled water. 5 µl of the mixture was added to a cleaned silicon chip and sonicated in acetone, ethanol, and distilled water for 5 min. Each solvent was flushed with water, blown dry, mounted on a stage with carbon paste, and then conducted at low beam energies (5–10 kV).

### Proteins markers characterization of exosomes

Total proteins were isolated from the exosomes following the manufacturer’s instructions for the total exosome RNA/protein isolation kit (Invitrogen™, UAB). Protein concentration was measured using the Bradford assay *via* a ^Jenway^Nanodrop. Exosomal protein markers, anti-PDCD6IP, anti-CD63, and anti-TSG101 (Wuhan Fine Biotech Co., Ltd., China) were detected using the western blotting technique and analyzed by a Gel Dox XR + Gel Documentation System (Bio-Rad Laboratories, Inc.). Six lanes for one sample were loaded.

### Exosome uptake efficiency

Labeling of exosomes by fluorescent dye: 10 and 20 µg protein equivalents of exosomes were resuspended in 100 µl PBS and then mixed with 100 µl of diluted PHK67 dye (1:1 v/v using diluents C) for 5 min. The mixture was diluted with 7.5 ml PBS and ultracentrifuged at 100,000*×g* for 90 min. Labeled exosomes (10 µg and 20 µg) were used to determine the efficiency of exosome uptake. For each cell line, in 6 well-plate, 50,000 cells/well were cultured in DMEM supplemented without FBS (starvation for 24 h) [24] and maintained at 37 °C in 5% CO_2_ until 80% confluence. After changing the media, labeled exosomes were added to the cells and incubated with complete media for 48 h. After extensive washing to remove any extracellularly labeled exosomes, the cells were examined under an immunofluorescence microscope to monitor exosome uptake efficiency. The experiment was repeated three times for each cell line.

### Cytotoxicity assay

The cytotoxic effects of AG490 and DOXO treatments were measured in the MDA-MB-231, HCC1806, and MCF7 cells using MTT assay. According to the ATCC protocol, 10,000 cells/well were seeded in 96-well plates and maintained at 37 °C in a 5% CO_2_ incubator until the cells reached 80% confluence. Drugs were added to the appropriate wells at different concentrations, supplemented with a complete media to a final volume of 200 µl, and then incubated for 48 h. After the incubation, 10 µl of MTT solution (5 mg/mL) was added to cells and incubated for 4 h at 37 °C. Control cells were ready as 50 µl of cells and 50 µl of MTT solution. After the incubation, 100 µl of dimethyl sulfoxide (DMSO) was added after removing the media. The optical density was determined at 590 nm by an ELISA reader (BioBioTek Company, UA). The count of living cells was recorded using trypan blue (1:1 ratio) to calculate the relative viability (% control). Relative viability was calculated with the equation as (experimental absorbance - background absorbance) / (absorbance of untreated controls -background absorbance) × 100%. Assuming the percentage of cell survival in the negative control was 100. The half-maximal inhibitory concentration (IC_50_) values were determined using relative viability measurements by Compusyn software (https://www.combosyn.com/). The experiment was repeated three times.

### Assessment of STAT3 signaling pathway inhibition

According to the ATCC protocol, utilized cell lines, TNBC and NTNBC, were treated with IC_50_ of AG490 drug. After 48 h, cells were gathered from treated (InST) and untreated (Un-InST) cells from each cell line. Total proteins were extracted from 1 × 10^5^ cells and measured by ^Jenway^nanodrop. Using the western blotting technique, the pan-STAT3 (STAT3 mAb, Wuhan Fine Biotech Co., Ltd., China), phosphor-STAT3 (Tyr705-STAT3 mAb, Cell Signaling Technology, USA) proteins, and GAPDH protein (Novus Biologicals, LLC, USA) as internal control, were determined to assess the activity of STAT3 signaling. The protein band intensities were analyzed using ImageLab software. Computational analysis was performed on a blot membrane containing the three cell lines, with at least two replicates per condition evaluated pre- and post-inhibition.

### Experimental design

The study enrolled TNBC and NTNBC cells and categorized them into eight groups. (1) Un-Treated group, cells without any treatment, (2) Exosomes (EXO) group, treated cells with exosomes, depending on the exosome uptake test, equivalent to 20 µg/protein, (3) Doxorubicin (DOXO) group; DOXO treated cells with IC50 of doxorubicin, (4) EXO-DOXO group, treated cells with 20 µg EXO and IC_50_ of DOXO, (5) InST-group, STAT3 inhibited cells treated with IC_50_ of AG490; STAT3 pathway inhibitor (6) InST-EXO group, InST-cells treated with 20 µg EXO, (7) InST-DOXO group: InST-cells treated with the IC_50_ of DOXO, and (8) InST-EXO-DOXO group: InST-cells treated with 20 µg EXO and IC_50_ of DOXO.

According to the experimental design, in 12-well plates, 50,000 cells/well were cultured in media without FBS (starvation for 24 h) [24] and maintained at 37 °C in 5% CO_2_. The media was changed after 24 h, with complete media, and incubated at 37 °C in 5% CO_2_ till the cells reached 80% confluence. For the Un-InST-treated cells, EXO and/or DOXO were added and incubated for 48 h. For InST cells, AG490 drug was added, and then cells were incubated for 48 h. After incubation, EXO and/or DOXO were added to cells after washing and then incubated at 37 °C in 5% CO_2_ for another 48 h. After the incubation, cell trypsinization was carried out by adding 0.2 ml of trypsin-EDTA, and the cells were subsequently harvested and centrifuged at 2000x*g* for 5 min and collected for downstream analysis. Viable cells were counted using trypan blue by adding 100 µl of premixed cell suspension to 100 µl of 0.4% trypan blue solution on a hemocytometer. The percentage of cell viability was calculated as “Cells/ml = $\:\frac{\text{n}\text{o}\:\text{o}\text{f}\:\text{t}\text{o}\text{t}\text{a}\text{l}\:\text{c}\text{e}\text{l}\text{l}\text{s}\:\text{c}\text{o}\text{u}\text{n}\text{t}\text{e}\text{d}}{\text{n}\text{o}\:\text{o}\text{f}\:\text{s}\text{q}\text{u}\text{a}\text{r}\text{e}\text{s}\:}$ × dilution factor × 10,000”.

### Migration assay

In a 12-well plate, 50,000 cells/1.5 ml of media were inoculated and cultured to 100% confluence. The cells were wounded by denuding a strip of the monolayer with a 200 µl pipette tip and then washed twice with PBS. According to the experimental design, Un-InST and InST cells were incubated with EXO and/or DOXO for 48 h at 37 °C in a 5% CO_2_ incubator. The rate of wound closure was assessed at a magnification of ×4 using a light microscope and analyzed using ImageLab software. The experiment was repeated three times.

### Colony assay

Un-InST and InST cells, in 12-well plates, 1000 cells/well were inoculated and allowed to grow at 37 °C and 5% CO_2_ incubator for 12 h until small colonies were observed. According to the experimental design, EXO and/or DOXO drugs were added to cells and incubated for 14 days, changing the media every 72 h. After they had incubated, the cells were fixed with 4% formaldehyde, stained with 0.1% crystal violet, incubated for 15 min, and then washed to remove excess staining. Images of the colonies were analyzed using ImageLab software. The experiment was repeated three times.

### Signaling pathways assessment

Total RNA was isolated from harvested cells using a GENEzol™ TriRNA Pure Kit (Geneaid Biotech Ltd., Taiwan). The extracted RNA was reverse transcribed to cDNA using a HiSenScript™ RH(-) cDNA Synthesis Kit (iNtRON-Biotechnology, Inc., Korea). cDNA was used for targeted gene detection in the Wnt signaling pathway (β-catenin, Cyclin D1, WNT1, and AXIN2) and in the Notch signaling pathway (NOTCH1, NOTCH2, JAG1, HES1, and DLL4) *via* relative quantitative polymerase chain reaction (qRT-PCR) using an Applied Biosystems™ 7500 Real-Time (Thermo Fisher Scientific, USA). β-ACTIN gene served as an internal control. Table 1 The PCR mixture was prepared with 10 µl of TOPreal™ master mix, 2 µl of cDNA, and 0.0.5µM of each primer, and then completed the volume to 20 µl. The PCR procedure was as follows: 95 °C for 10 min; 45 cycles of 95 °C for 15 s and 56 °C for 15 s; and 72 °C for 30 min. The mRNA gene expression levels were calculated for six replicas for each treatment using the comparative (2^−ΔΔCT^) method.

**Table 1:** Primers sequence of targeted genes, WNT and Notch signaling pathways

Gene	Primer sequence (5′-3′)	Accession number
WNT signaling pathways		
β–Catenin	F: 5’- GATTTGATGGAGTTGGACATGG − 3’ R: 5’-TGTTCT TGAGTGAAGGACTGAG-3’	NM_001330729.2
Cyclin D1	F: 5’-TGCATGTTCGTGGCCTCTAA-3’ R: 5’-TCGGTGTAGATGCACAGCTT-3’	NM_053056.3
WNT 1	F: 5’- GCAGTGACAACATCGATTTTGG − 3’ R: 5’- TCTTGGCGCATCTCAGAGAAC-3’	NM_005430.4
AXIN2	F: 5’-TACACTCCTTATT-GGGCGATCA-3′ R: 5-’AAGTCTGG-CTCGTTCTCAGTG-3′	NM_001363813
Notch signaling pathway		
NOTCH1	F: 5’-CCGCCTTTGTGCTTCTGTT-3’ R: 5’-TCCTCCTCTTCCTCGCTGTT-3’	NM_017617
NOTCH2	F: 5’-GATGCCACCTGAACAACTGC-3′ R: 5’-TGACAACAGCAACAGCAAGG-3′	NM_024408
JAG1	F: 5’-GATCCTGTCCATGCAGAACG-3’ R: 5’-GGATCTGATACTCAAAGTGG-3’	NM_000214
HES1	F: 5’-GACAGCATCTGAGCACAGAAATG-3’ R: 5’-GTCATGGCATTGATCTGGGTCAT-3’	NM_005524
DLL4	F: 5’-CCAACTATGCTTGTGAATGTCC-3’ R: 5’-TGTGGAGAGGTCGGTGTAGC-3’	NM_019074
β-ACTIN	F: 5’-TGGCACCACACCTTCTACAATGAGC-3’ R: 5’-GCACAGCTTCTCCTTAATGTCACGC-3’	NM_001101

**Table 2:** Descriptive analysis of the expression levels of WNT pathway-targeted genes (fold changes) in MDA-MB-231, HCC1806 and MCF7 cells after EXO and/or DOXO administration for 48 h before and after STAT3 pathway Inhibition

Genes	β-catenin	Cyclin D1		WNT1		AXIN2	
Groups	Mean ± S.E.	Mean ± S.E.		Mean ± S.E.		Mean ± S.E.	
MDA-MB-231							
Un-Treated	1.03 ± 0.04	0.99 ± 0.03		1.00 ± 0.02		1.00 ± 0.03	
EXO	**4.33 ± 0.76** ^*^	**2.18 ± 0.17** ^*^		**1.92 ± 0.06** ^*^		**1.98 ± 0.25** ^*^	
DOXO	**4.14 ± 0.29** ^*^	**1.60 ± 0.10** ^*^		**0.64 ± 0.04** ^*^		**1.75 ± 0.08** ^*^	
EXO-DOXO	**0.74 ± 0.06** ^**#**^	**1.00 ± 0.06** ^**#**^		**0.90 ± 0.03** ^**#**^		**1.03 ± 0.14** ^**#**^	
InST	0.14 ± 0.01	0.32 ± 0.05		0.16 ± 0.09		0.77 ± 0.01	
InST-EXO	**0.34 ± 0.04** ^**¥**^	**1.26 ± 0.08** ^**¥**^		**0.50 ± 0.01** ^**¥**^		**0.27 ± 0.03** ^**¥**^	
InST-DOXO	**1.87 ± 0.11** ^**#**^	**1.21 ± 0.06** ^**#**^		**0.20 ± 0.03** ^**#**^		**0.83 ± 0.04** ^**#**^	
InST-EXO-DOXO	**0.23 ± 0.04** ^**Ѱ, €**^	1.47 ± 0.15		**0.41 ± 0.04** ^**Ѱ, €**^		**0.67 ± 0.04** ^**Ѱ, €**^	
HCC1806							
Un-Treated	0.99 ± 0.04	0.95 ± 0.06		1.06 ± 0.04		1.00 ± 0.06	
EXO	**1.53 ± 0.11** ^*^	1.05 ± 0.08		1.08 ± 0.09		1.04 ± 0.04	
DOXO	0.94 ± 0.02	**1.40 ± 0.09** ^*^		**0.74 ± 0.05** ^*^		1.20 ± 0.02	
EXO-DOXO	**1.41 ± 0.12** ^**#**^	1.41 ± 0.05		**1.16 ± 0.04** ^**#**^		1.23 ± 0.09	
InST	1.05 ± 0.02	1.20 ± 0.02		1.49 ± 0.15		1.52 ± 0.08	
InST-EXO	**0.96 ± 0.05** ^**¥**^	1.05 ± 0.02		0.99 ± 0.05		1.16 ± 0.03	
InST-DOXO	0.94 ± 0.03	1.21 ± 0.02		**1.07 ± 0.01** ^**#**^		1.12 ± 0.03	
InST-EXO-DOXO	**1.00 ± 0.04** ^**Ѱ**^	1.37 ± 0.16		1.19 ± 0.07		**0.77 ± 0.01** ^**Ѱ, €**^	
MCF7							
Un-Treated	1.02 ± 0.11	1.00 ± 0.06		1.03 ± 0.06		1.00 ± 0.02	
EXO	**1.44 ± 0.03** ^*^	**1.77 ± 0.14** ^*^		0.93 ± 0.01		1.06 ± 0.03	
DOXO	0.88 ± 0.09	**1.49 ± 0.10** ^*^		1.20 ± 0.08		1.07 ± 0.04	
EXO-DOXO	**1.58 ± 0.10** ^**#**^	1.56 ± 0.16		**1.59 ± 0.04** ^**#**^		**0.89 ± 0.0** ^**#**^	
InST	1.36 ± 0.05	1.63 ± 0.09		1.19 ± 0.04		1.22 ± 0.08	
InST-EXO	1.29 ± 0.08	1.56 ± 0.08		**1.62 ± 0.07** ^**¥**^		1.14 ± 0.06	
InST-DOXO	**2.01 ± 0.21** ^**#**^	**1.09 ± 0.14** ^**#**^		**1.45 ± 0.05** ^**#**^		**2.09 ± 0.06** ^**#**^	
InST-EXO-DOXO	**1.40 ± 0.12** ^**€**^	1.40 ± 0.11		**0.87 ± 0.04** ^**Ѱ ,e**^		**1.34 ± 0.04** ^**Ѱ, €**^	

^*^ statistically significant compared to those of the untreated control groups

^¥^ Statistically significant difference compared to the EXO-treated group

^#^ Statistically significant difference compared to the DOXO-treated group

^Ѱ^ Statistically significant difference compared to the EXO-DOXO-treated group

^€^ Statistically significant difference compared to the InST-DOXO-treated group

p values ≤ 0.05 were considered to indicate statistical significance

**Table 3:** Descriptive analysis of the expression levels of Notch pathway-targeted genes (fold changes) in MDA-MB-231, HCC1806 and MCF7 cells after EXO and/or DOXO administration for 48 h before and after STAT3 pathway Inhibition

Genes	NOTCH1	NOTCH2	JAG1	HES1	DLL4
Groups	Mean ± S.E.	Mean ± S.E.	Mean ± S.E.	Mean ± S.E.	Mean ± S.E.
MDA-MB-231 cells					
Untreated	1.02 ± 0.07	1.04 ± 0.06	1.08 ± 0.04	1.07 ± 0.05	1.00 ± 0.01
EXO	1.10 ± 0.02	**6.33 ± 0.72** ^*^	**3.85 ± 0.12** ^*^	**3. 5 ± 0.35** ^**a8**^	**1.87 ± 0.25** ^*^
DOXO	1.07 ± 0.04	**6.05 ± 0.20** ^*^	**3.01 ± 0.08** ^*^	**3.3 ± 0.25** ^*^	**1.58 ± 0.14** ^*^
EXO-DOXO	1.03 ± 0.05	6.69 ± 1.36	**2.06 ± 0.07** ^**#**^	**1.91 ± 0.04** ^**#**^	**1.03 ± 0.01** ^**#**^
InST	0.42 ± 0.04	0.24 ± 0.01	0.53 ± 0.10	0.53 ± 0.08	1.07 ± 0.03
InST-EXO	**0.83 ± 0.05** ^**¥**^	**0.23 ± 0.01** ^**¥**^	**1.05 ± 0.01** ^**¥**^	**0.39 ± 0.03** ^**¥**^	**0.28 ± 0.01** ^**¥**^
InST-DOXO	**0.79 ± 0.04** ^#^	**0.94 ± 0.03** ^#^	**2.15 ± 0.15** ^#^	**0.43 ± 0.07** ^#^	**0.90 ± 0.02** ^#^
InST-EXO-DOXO	**0.32 ± 0.05** ^**Ѱ,€**^	**0.45 ± 0.11** ^**Ѱ,€**^	**1.19 ± 0.03** ^**e**^	**0.39 ± 0.1** ^**Ѱ**^	**0.54 ± 0.03** ^**Ѱ,€**^
HCC1806 cells					
Untreated	1.00 ± 0.05	1.00 ± 0.02	1.08 ± 0.05	1.00 ± 0.02	1.07 ± 0.01
EXO	**1.45 ± 0.03** ^*^	**1.65 ± 0.05** ^*^	1.01 ± 0.03	**1.52 ± 0.03** ^*^	1.01 ± 0.04
DOXO	**1.44 ± 0.01** ^*^	1.06 ± 0.04	**0.68 ± 0.01** ^*^	1.05 ± 0.02	1.10 ± 0.03
EXO-DOXO	1.48 ± 0.01	**1.67 ± 0. 34** ^**#**^	**1.59 ± 0.12** ^**#**^	1.17 ± 0.07	1.01 ± 0.03
InST	0.59 ± 0.01	1.04 ± 0.01	0.71 ± 0.03	0.83 ± 0.04	1.11 ± 0.02
InST-EXO	**0.51 ± 0.05**	**0.75 ± 0.04** ^**¥**^	**0.40 ± 0.07** ^**¥**^	**1.02 ± 0.02** ^**¥**^	**0.84 ± 0.02** ^**¥**^
InST-DOXO	1.56 ± 0.05	**0.69 ± 0.14** ^**#**^	0.64 ± 0.03	1.06 ± 0.01	0.95 ± 0.02
InST-EXO-DOXO	**0.86 ± 0.03** ^**Ѱ,€**^	**0.60 ± 0.03** ^**Ѱ**^	**0.65 ± 0.04** ^**Ѱ**^	1.08 ± 0.03	1.01 ± 0.06
MCF7 cells					
Untreated	1.00 ± 0.04	1.00 ± 0.06	1.01 ± 0.03	1.0 ± 0.05	1.04 ± 0.08
EXO	1.20 ± 0.05	1.30 ± 0.26	1.22 ± 0.01	1.12 **±** 0.01	1.04 ± 0.01
DOXO	0.91 ± 0.01	1.13 ± 0.07	1.21 ± 0.11	**0.71 ± 0.05** ^*^	**0.70 ± 0.06** ^*^
EXO-DOXO	**2.07 ± 0.39** ^**#**^	**2.16 ± 0.12** ^**#**^	**1.80 ± 0.02** ^**#**^	1.30 ± 0.03	1.17 ± 0.01
InST	2.32 ± 0.11	2.16 ± 0.09	1.73 ± 0.07	1.24 **±** 0.05	1.12 ± 0.03
InST-EXO	**2.51 ± 0.49** ^**¥**^	**2.71 ± 0.36** ^**¥**^	**1.75 ± 0.09** ^**¥**^	**1.56 ± 0.21** ^**¥**^	**1.53 ± 0.17** ^**¥**^
InST-DOXO	**2.90 ± 0.71** ^**#**^	**2.06 ± 0.20** ^**#**^	**2.38 ± 0.22** ^**#**^	**1.29 ± 0.07** ^**#**^	**1.65 ± 0.10** ^**#**^
InST-EXO-DOXO	**4.10 ± 0.18** ^**Ѱ, €**^	2.05 ± 0.10	2.10 ± 0.08	1.37 ± 0.03	**2.70 ± 0.13** ^**Ѱ, €**^

* statistically significant compared to those of the untreated control groups

^¥^ Statistically significant difference compared to the EXO-treated group

^#^ Statistically significant difference compared to the DOXO-treated group

^Ѱ^ Statistically significant difference compared to the EXO-DOXO-treated group

^€^ Statistically significant difference compared to the InST-DOXO-treated group

p values ≤ 0.05 were considered to indicate statistical significance

### Statistical analysis

Statistical analysis was performed using SPSS version 22.0. The Mann–Whitney U test, a nonparametric method, compared the means of various biochemical parameters between groups, with p values ≤ 0.05 considered statistically significant.

## Results

### Size characterization of exosomes

**Fig. 2:**
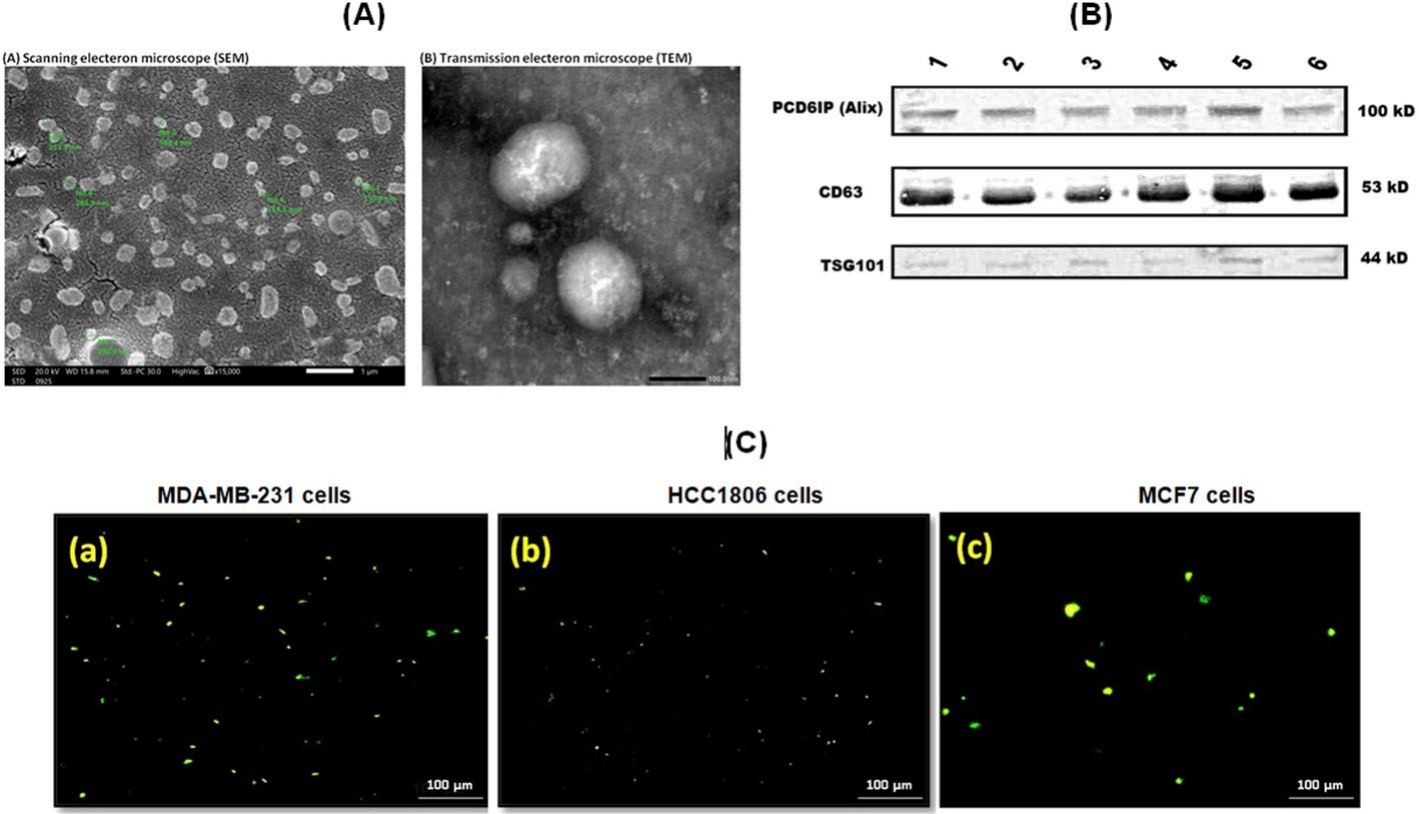
**A** Exosome characterization using a JSM-5300 scanning electron microscope (SEM), magnification: 15,000x and Transmission electron microscope (TEM), Scale: 500 nm. Exosomes have a biconcave shape and are of different sizes (100–300 nm). **B** Exosomal surface marker protein detection via western blotting. Cropped blotting image: Lanes (1–6): exosomes isolated from serum TNBC sample. The results were analyzed using a Gel DoxXR + Gel Documentation System (Bio-Rad). CD63 protein was highly expressed compared to the PCD6IP (Alix) and TSG101 proteins. The original blot was presented in Supplementary Fig. 1 & Fig. 3. **C** Fluorescence microscopy images of PKH67-labeled exosome uptake in the **a** MDA-MB-231, **b** HCC1806 and **c** MCF7 cell lines using a Labomed Triular inverted fluorescence microscope (Model 400), lens 40x. Scale bar: 100 μm

SEM and TEM images of exosomes isolated from the serum of a TNBC patient revealed biconcave shapes of different sizes (100–300 nm), and large subclasses were predominant. Figure 2A.

### Surface proteins characterization of exosomes

Western blotting analysis revealed that the isolated exosomes were positive for CD63, PCD6IP (Alix), and TSG101, indicating the presence of exosomal surface marker proteins. The intensity of the CD63 protein surface marker was greater than that of the PCD6IP (Alix) and TSG101 proteins. Figure 2B.

### Exosome uptake efficiency

Fluorescence microscopy images of PKH67 green fluorescent-labeled EXO demonstrated that exosomal uptake was more efficient in TNBC cell lines, with MDA-MB-231 cells showing slightly higher uptake than HCC1806 cells, compared to the NTNBC cell line (MCF7 cells). Figure 2C.

### Cytotoxicity test

**Fig. 3:**
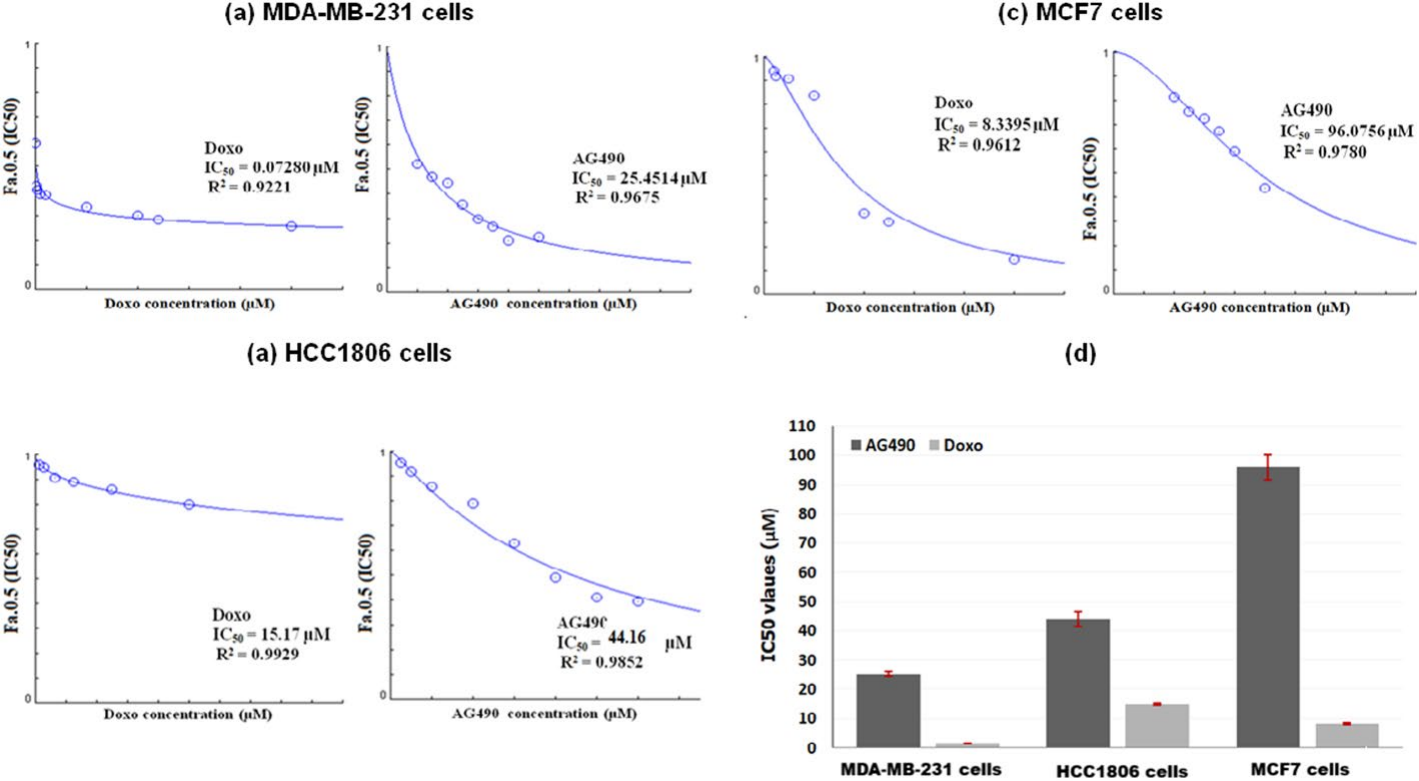
Half maximal inhibitory concentration (IC50) curves of AG490 and DOXO treatments against **a** MDA-MB-231, **b** HCC1806, and **c** MCF7 cells after 48 h. **d** Histograms showing the half maximal inhibitory concentration (IC50) values. Six replicas were used for each experiment, analysis was done with SPSS. R2 = linear correlation coefficient, Bars represent the standard error (S.E)

AG490 and DOXO treatments for 48 h inhibited the growth of the TNBC and NTNBC cell lines compared to untreated cells with significantly different effects. The IC_50_ values of AG490 against MDA-MB-231, HCC1806, and MCF-7 cells were 25.4 ± 0.009, 44.16 ± 0.026 and 96.07 ± 0.042 µM, respectively. The IC_50_ values of DOXO against MDA-MB-231, HCC1806, and MCF-7 cells MCF-7 cells were 1.72 ± 0.001, 15.17 ± 0.003 and 8.34 ± 0.009 µM, respectively. Results showed that MDA-MB-231 cells exhibited greater sensitivity to AG490 and DOXO than the other two cell lines. Figure 3.

### Assessment of STAT3 signaling pathway

**Fig. 4:**
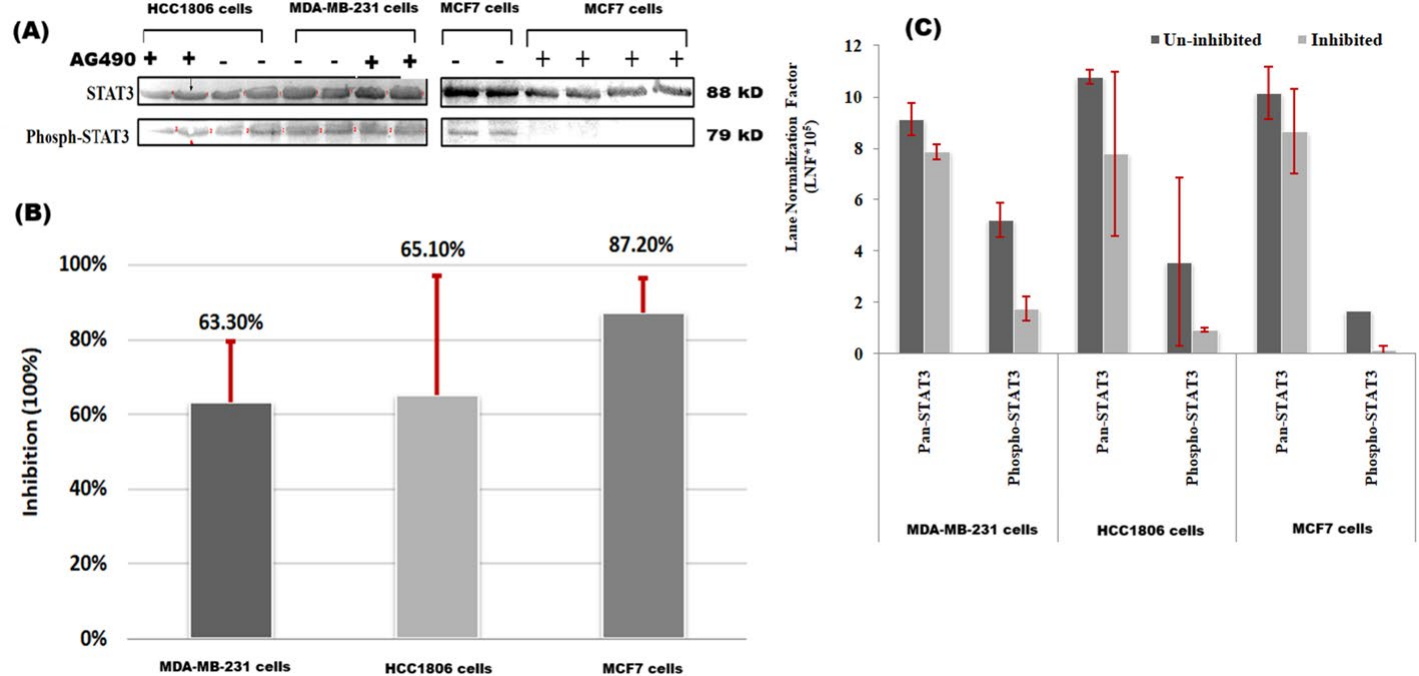
**A**Representative cropped images of western blots before (-) and after (+) STAT3 pathway inhibition with AG490 inhibitor were analyzed using a Gel Dox XR + Gel Documentation System. System (Bio-Rad). Original blot was presented in supplementary Fig. 2. **B** Plots showing the inhibitory effect (%) after 48 h of incubation. **C** Plots of the Lane Normalization Factor (LNF) for each cell line

The degree of STAT3 signaling inhibition using AG490 treatment was assessed using western blotting. The imaging analysis revealed weak p-STAT3 expression compared to pan-STAT3 expression after AG490 treatment of MDA-MB-231, HCC1806, and MCF7 cells. Figure 4A A Comparison of the percentage of inhibition revealed that compared with MDA-MB-231 (63.3 ± 0.16%) and HCC1806 (65.1 ± 0.32%) cells, MCF7 cells were the most inhibited (87.2 ± 0.09%). Figure 4B As shown in Fig. 4C, there was a marked difference in the response of different breast cancer cells to the AG490 inhibitor.

### Cell morphology

**Fig. 5:**
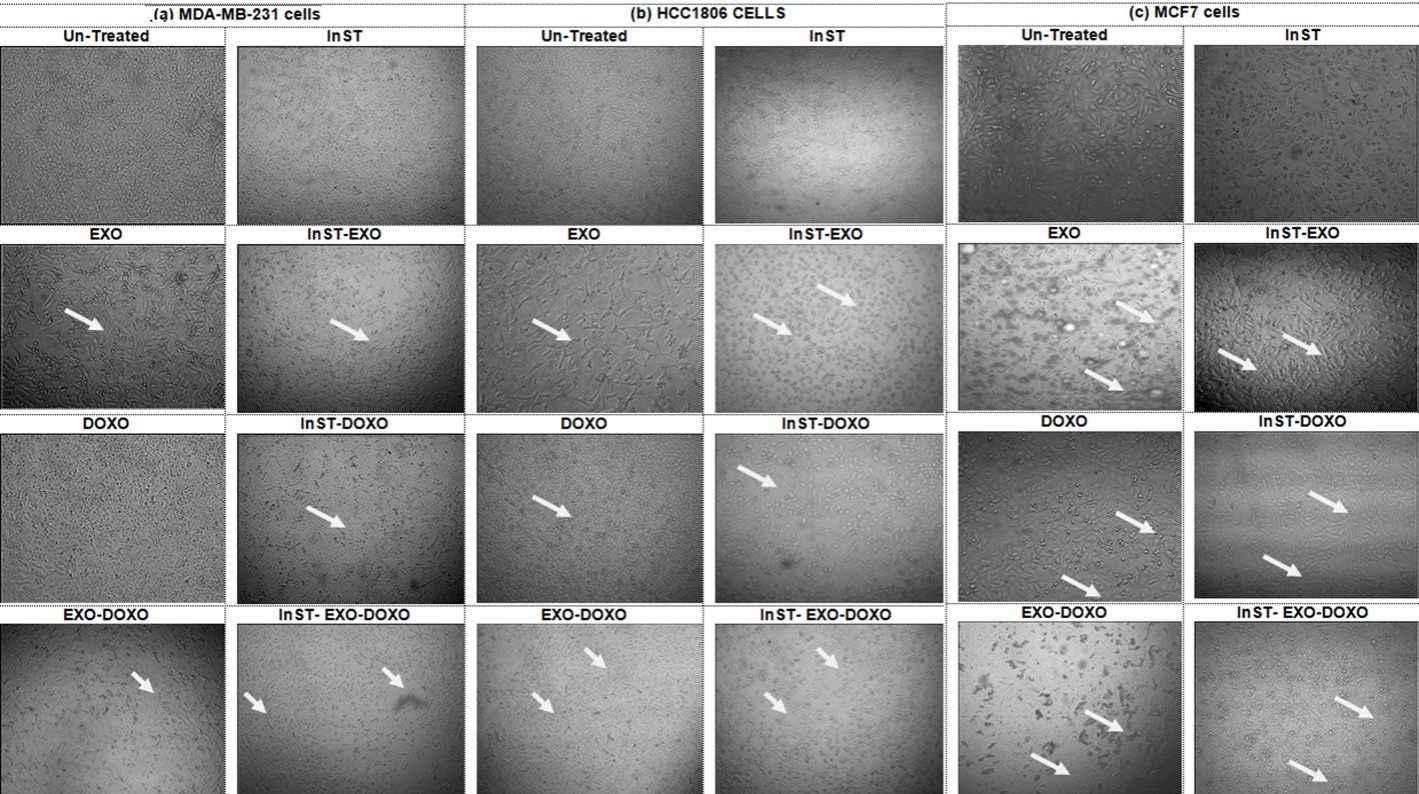
Morphological alterations in **A** TNBC cell lines, MDA-MB-231, HCC1806, and **B** NTNBC cell line, MCF7, after EXO and/or DOXO treatment for 48 h before and after STAT3 pathway inhibition

The impact of STAT3 pathway inhibition on the morphological changes of TNBC cells, MDA-MB-231 and HCC1806, and NTNBC cells, MCF7, examined under the effect of EXO and/or DOXO treatment and illustrated in Fig. 5. Examination of MDA-MB231 and HCC1806 cells after treatment with EXO revealed an elongated spindle-shaped structure of epithelial cells. However, MCF7 cells treated with EXO formed aggregates and clumps compared to untreated cells. Examinations of MDA-MB-231 and MCF7 cells treated with DOXO revealed the presence of rounded detached cells and debris, whereas the effect of DOXO was more pronounced in MCF7 cells than in MDA-MB-231 cells. However, no morphological changes in the HCC1806 cells were observed after treatment with DOXO compared to those in the untreated cells. However, compared with DOXO-treated cells, TNBC cells treated with EXO-DOXO had more elongated spindle**-**shaped structures. Moreover, compared to DOXO-treated cells, NTNBC cells treated with EXO-DOXO showed cellular aggregates and clumps.

Treatment of InST-MDA-MB-231 cells with EXO reduced the frequency of spindle-shaped cells compared to EXO treatment only. However, adding EXO to InST-HCC1806 cells showed small circular vacuoles and shrunk cells relative to EXO treatment. In InST-MCF7 cells, EXO addition eliminated cellular aggregates and clumps observed in EXO-treated cells. InST-TNBC and InST-NTNBC cells treated with DOXO showed no microscopic changes, but a reduction in viable cells was noted compared to DOXO treatment only. The EXO-DOXO combination did not induce morphological changes in InST-MDA-MB-231 cells compared to EXO-DOXO treatment, although elongated spindle shapes were observed for epithelial cells compared to InST-DOXO treatment. InST-HCC1806 cells still showed small circular vacuoles and shrunk cells with EXO-DOXO treatment compared to EXO-DOXO and InST-DOXO treatments. InST-MCF7 cells treated with EXO-DOXO exhibited the disappearance of cellular aggregates and clumpy formations relative to EXO-DOXO-treated cells, and small circular vacuoles were present compared to InST-DOXO treated cells.

### Migration assay

**Fig. 6:**
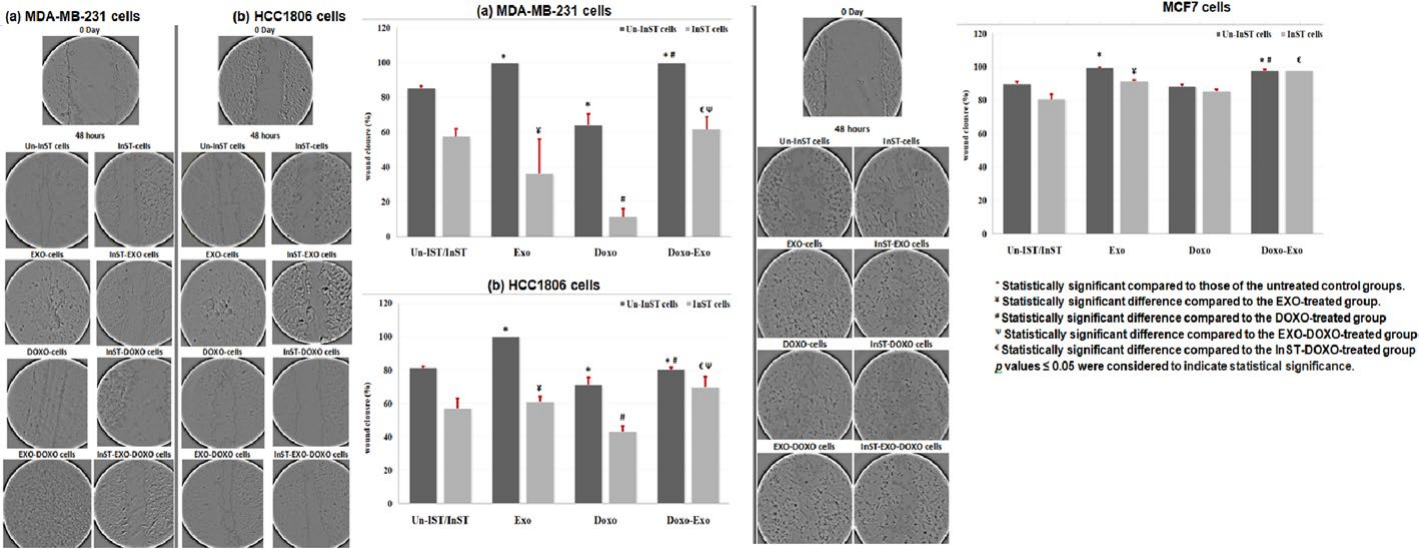
Microscopy images of the migration assay under an inverted microscope after EXO and/or DOXO treatment for 48 h before and after STAT3 pathway inhibition in **A** TNBC cell lines, MDA-MB-231, HCC1806, and **B** TNBC cell line, MCF7. Three replicas were used for each experiment and analysis was done using ImagJ software

The migration was assessed in TNBC cells, MDA-MB-231 and HCC1806, and NTNBC cells, MCF7, following STAT3 pathway inhibition, with treatment involving EXO and/or DOXO. Figure 6.

Treatment of TNBC and NTNBC cells with EXO completely healed the tumor closure area (100%) compared to untreated cells. A distinct aggressive clump in the wound area was observed in TNBC and MDA-MB-231 cells, with a lesser extent in HCC1806 cells. However, compared with no treatment, DOXO treatment of TNBC cells resulted in a significantly decreased closure area in MDA-MB-231 cells (64 ± 6.5%), HCC1806 (71 ± 4.6%) cells, with an insignificant decrease in NTNBC cell line, MCF7 (88 ± 1.1%) cells. Interestingly, treatment of TNBC cells and NTNBC cells with EXO-DOXO significantly increased the closure area of MDA-MB-231 (100%), HCC1806 (80 ± 1.1%), and MCF7 cells (98 ± 0.46%) compared with that of DOXO-treated cells.

In InST- cells, treatment of InST-TNBC cells with EXO also significantly decreased cell migration (36 ± 19.9% and 61 ± 3.16% in MDA-MB-231 and HCC1806 cells, respectively) compared to that in EXO-treated cells. Treatment of InST-NTNBC cells with EXO minimally affected cell migration (92 ± 0.56%) compared to EXO-treated cells. Moreover, the inhibitory effect of DOXO on cell migration was more pronounced in TNBC cells than in NTNBC cells when compared to the corresponding DOXO-treated cells. Thus, treatment of InST cells with DOXO resulted in a significant reduction in the closure area of TNBC cells, MDA-MB-231 (64 ± 6.5% to 11 ± 4.7%), and HCC1806 cells (71 ± 4.5% to 43 ± 3.3%) than in NTNBC cells (88 ± 1.1% to 85 ± 1.0%). Treatment of InST-TNBC cells with EXO-DOXO markedly reduced the wound area in MDA-MB-231 (62 ± 6.9%) and HCC1806 (70 ± 6.2%) cells. Moreover, there was no difference in the growth of MCF7 cells (98 ± 0.12%) compared to that of the corresponding EXO-DOXO-treated cells (MDA-MB-231 100 ± 0.0%, HCC1806 80 ± 1.1%, and MCF7 98 ± 0.46%). In contrast, compared to INST-DOXO treated cells, an obvious increase in the wound area was also observed in TNBC (MDA-MB-231; from 11 ± 4.7% to 62 ± 6.9% and HCC1806; from 43 ± 3.3% to 70 ± 6.2%) and NTNBC cells (MCF7; from 85 ± 1.0% to 98 ± 0.12%).

### Colony assay

**Fig. 7:**
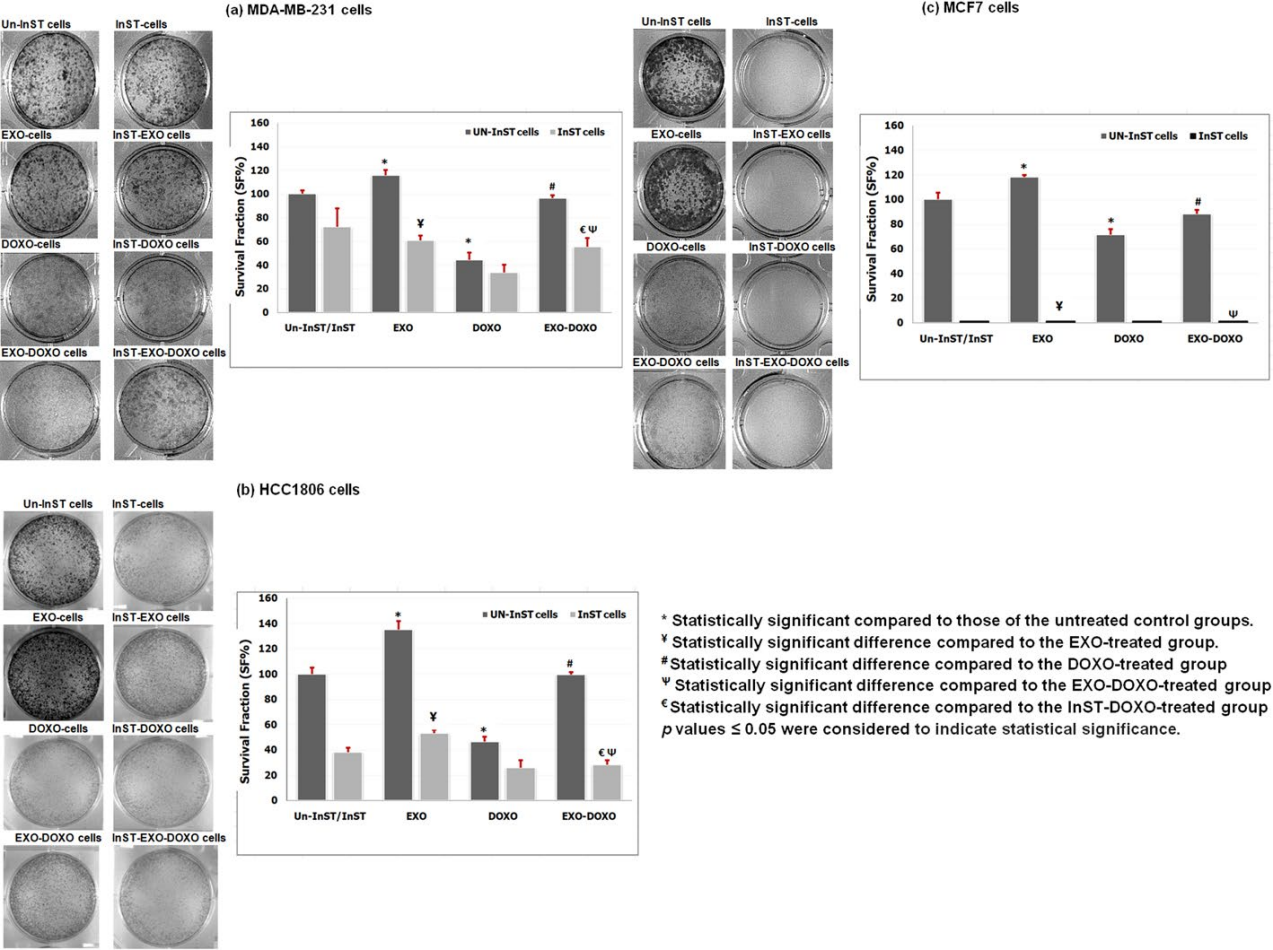
Colony formation assay in (**A**) TNBC cell lines, MDA-MB-231, HCC1806, and (**B**) NTNBC cell line, MCF7, after EXO and/or DOXO treatment for 48 h before and after STAT3 pathway inhibition. Three replicas were used for each experiment and analysis was done using ImagJ software

The effect of STAT3 pathway inhibition on colony formation in TNBC cells, MDA-MB-231 and HCC1806, and NTNBC cells, MCF7, under EXO and/or DOXO treatment was shown in Fig. 7.

EXO treatment increased tumorigenic effects in TNBC and NTNBC cells, as evidenced by a significantly higher percentage of colonies comparing untreated cells. HCC1806 cells showed the greatest colonization at 135 ± 6.8%, followed by MCF7 cells at 118 ± 2.0% and MDA-MB-231 cells at 116 ± 4.8%. In contrast, DOXO treatment exhibited antitumor effects across MDA-MB-231, HCC1806, and MCF7 cells, with reductions in cell colonization of 44 ± 6.4%, 46 ± 4.1%, and 71 ± 4.5%, respectively, compared to untreated cells. EXO-DOXO treatment resulted in increased cell colonization relative to DOXO treatment alone, with MCF7 cells at 88 ± 3.3%, MDA-MB-231 cells at 96 ± 2.4%, and HCC1806 cells at 99 ± 1.9%. These results suggest that EXO is more effective at promoting colonization in HCC1806 than in MDA-MB-231 or MCF7 cells.

Further, EXO-treated InST-TNBC cells exhibited decreased colonization in HCC1806 (61 ± 4.2%) and MDA-MB-231 cells (53 ± 2.7%). Interestingly, no colonies were observed in InST-NTNBC MCF7 cells. The cytotoxic impact of DOXO was more significant in InST-treated cells, leading to reduced colonization in InST-HCC1806 cells (26 ± 5.9%) and InST-MDA-MB-231 cells (34 ± 6.5%), with no colony formation in InST-MCF7 cells. Treatment with EXO-DOXO in MDA-MB-231 and HCC1806 cells reduced colonization compared to EXO-DOXO-treated cells, with HCC1806 cells at 28 ± 3.5% and MDA-MB-231 cells at 56 ± 7.5%. InST-MCF7 cells treated with EXO-DOXO showed no colonization, while MDA-MB-231 cells increased from 34 ± 6.5% to 56 ± 7.5% and HCC1806 cells from 26 ± 5.9% to 28 ± 3.5% compared to InST-DOXO-treated cells.

### Signaling pathways assessment

#### WNT signaling pathway

**Fig. 8:**
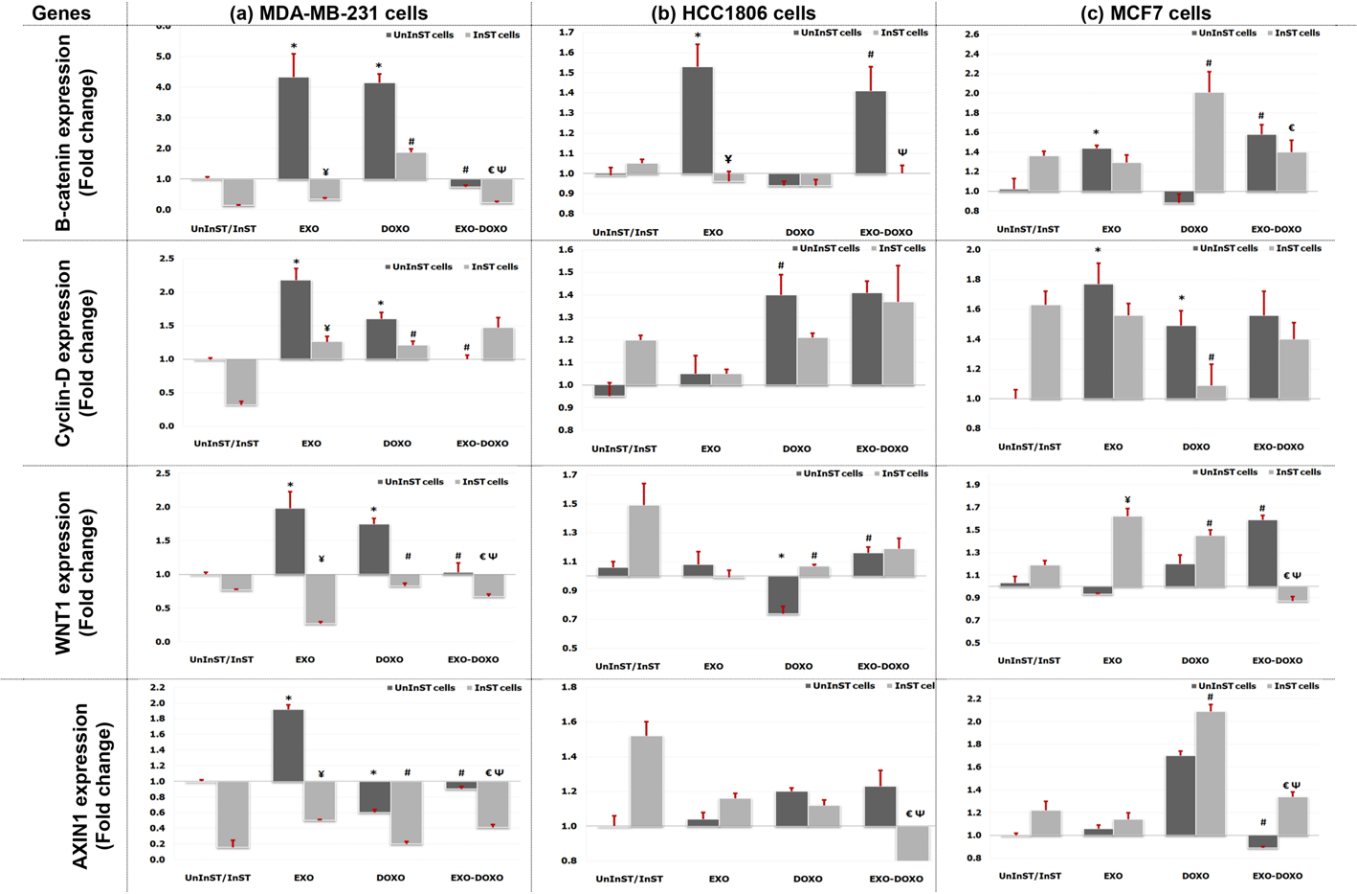
Expression analysis of targeted genes in the Wnt signaling pathways in MDA-MB-231, HCC1806, and MCF7 cells after 48 h of EXO and/or DOXO treatment, both before and after STAT3 pathway inhibition. Six replicas were used for each experiment and analyzed using the SPSS program

EXO treatment significantly increased the expression of β-catenin, Cyclin D1, WNT1, and AXIN2 in MDA-MB-231 cells compared to untreated cells. In HCC1806 cells, only β-catenin expression was significantly increased with EXO treatment. In MCF7 cells, EXO treatment significantly increased the expression of β-catenin and Cyclin D1. Conversely, DOXO treatment in MDA-MB-231 cells increased β-catenin, Cyclin D1, and AXIN2 but decreased WNT1 expression. In HCC1806 cells, DOXO treatment significantly increased only Cyclin D1, while WNT1 expression decreased. MCF7 cells treated with DOXO showed a significant increase in Cyclin D1 expression without affecting other genes expression. When comparing EXO-DOXO to DOXO treatment in MDA-MB-231 cells, there was a notable decrease in β-catenin, Cyclin D1, and AXIN2, alongside a significant increase in WNT1 expression. EXO-DOXO also significantly raised β-catenin and WNT1 levels in HCC1806 and MCF7 cells, with no changes in AXIN2 expression in HCC1806, while MCF7 exhibited significantly lower AXIN2 levels than in DOXO-treated cells. Table 2 & Fig. 8.

In InST cells, compared to EXO treatment, EXO treatment in InST-MDA-MB-231 cells notably reduced β-catenin, Cyclin D1, WNT1, and AXIN2 expression. In InST-HCC1806 cells, β-catenin expression was the only significantly decreased gene. On the other hand, WNT1 expression increased in InST-EXO-treated MCF7 cells compared to EXO treatment. With DOXO treatment, InST-MDA-MB-231 cells showed decreased β-catenin, Cyclin D1, WNT1, and AXIN2 expression, while InST-HCC1806 cells exhibited a significant increase in the expression of WNT1 only. In InST-DOXO-treated MCF7 cells, the expression of β-catenin, WNT1, and AXIN2 significantly increased, but Cyclin D1 expression decreased. Compared to EXO-DOXO, InST-MDA-MB-231 cells showed significant reductions in β-catenin, WNT1, and AXIN2 expression. InST-HCC1806 cells experienced decreased β-catenin and AXIN2 expression with EXO-DOXO treatment, whereas WNT1 expression decreased, and AXIN2 expression increased in InST-MCF7 cells compared to EXO-DOXO treatment. Additionally, InST-MDA-MB-231 cells treated with EXO-DOXO had lower β-catenin and AXIN2 expression alongside increased WNT1 expression compared to InST-DOXO cells. In InST-HCC1806 cells, AXIN2 expression also significantly decreased. In contrast, InST-MCF7 cells showed significant reductions in β-catenin, WNT1, and AXIN2 expression with EXO-DOXO treatment. Table 2 & Fig. 8.

### Notch signaling pathway

**Fig. 9:**
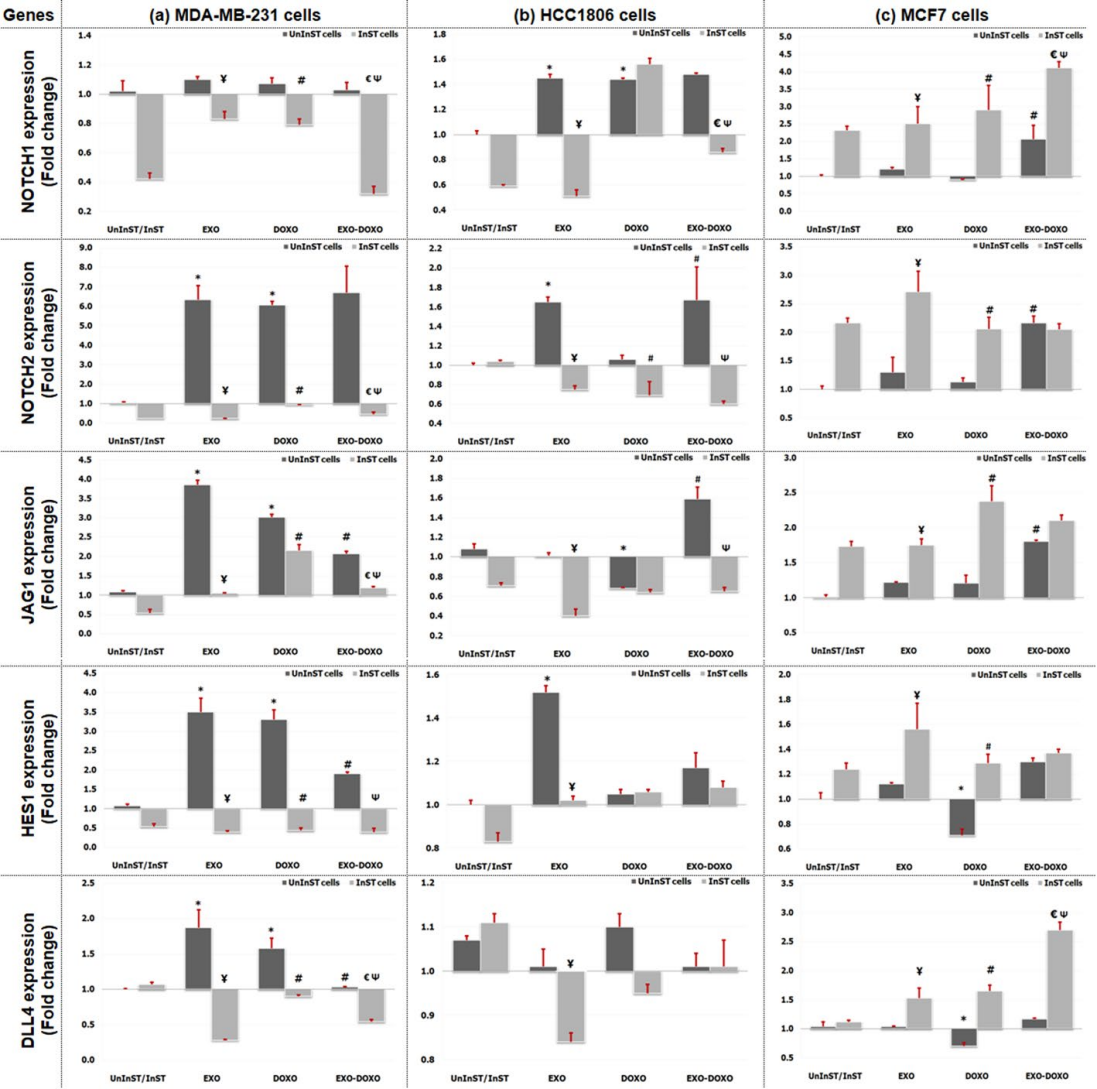
Expression analysis of targeted genes in the Notch signaling pathways; **A**)Notch1, Notch2, and JAG1 **B** HES1 and DLL4, in MDA-MB-231, HCC1806, and MCF7 cells after 48 h of EXO and/or DOXO treatment, both before and after STAT3 pathway inhibition. Six replicas were used for each experiment and analyzed using the SPSS program

Compared with no treatment, treatment with EXO significantly increased NOTCH2, JAG1, HES1, and DLL4 expression in MDA-MB-231 cells. However, in HCC1806 cells, treatment significantly increased NOTCH1, NOTCH2 and HES1 expression. Moreover, in MCF7 cells, no significant changes in the expression of Notch pathway signaling genes were observed. On the other hand, DOXO treatment of MDA-MB-231 cells significantly increased NOTCH2, JAG1, HES1, and DLL4 expression. However, after DOXO treatment, only NOTCH1 expression was significantly increased in HCC1806 cells; in contrast, JAG1 expression was significantly decreased. However, DOXO treatment of MCF7 cells significantly decreased HES1 and DLL4 expression. Compared with DOXO treatment, EXO-DOXO treatment of MDA-MB-231 cells significantly reduced the expression of JAG1, HES1, and DLL4. However, in HCC1806 cells, treatment significantly increased NOTCH2 and JAG1 expression. However, in MCF7 cells, EXO-DOXO treatment significantly increased NOTCH1, NOTCH2, and JAG1 expression compared to DOXO-treated cells. Table 3 & Fig. 9.

In InST cells, after treatment of TNBC cells with EXO, MDA-MB-231 and HCC1806 cells exhibited significantly lower expression levels of all the genes in the Notch signaling pathway than the corresponding cells without STAT3 inhibition. In contrast, compared with no STAT3 inhibition, EXO treatments significantly increased the expression of all genes in the Notch signaling pathway in InST-NTNBC cells and MCF7 cells. Additionally, compared with no STAT3 inhibition, DOXO treatment significantly decreased the expression of all genes in the Notch signaling pathways in InST-MDA-MB-231 cells. In InST-HCC1806 cells, DOXO treatment decreased NOTCH2 expression only. In contrast, DOXO treatment of InST-MCF7 cells increased the expression of all the target genes of the Notch signaling pathway compared to that in DOXO cells without DOXO inhibition. Table 3 & Fig. 9.

Exosomes in combination with DOXO treatment significantly decreased the expression of genes associated with the Notch signaling pathway in InST-MDA-MB-231 cells compared to that in the corresponding cells without STAT3 inhibition. However, in InST-HCC1806 cells, EXO-DOXO treatment significantly decreased NOTCH1, NOTCH2, and JAG1 expression. In contrast, in InST-MCF7 cells, NOTCH1 and DLL4 expression was significantly increased. Additionally, compared to those in InST-DOXO cells, EXO-DOXO treatment of InST-MDA-MB-231 cells significantly decreased NOTCH1, NOTCH2, HES1, and DLL4 expression. Moreover, in InST-HCC1806 cells, NOTCH1 expression was significantly decreased; however, in InST-MCF7 cells, Notch1 and DLL4 expression was significantly increased. Table 3 & Fig. 9.

## Discussion

The SEM and TEM analysis aligns with previous studies demonstrating that large exosomes exhibit a distinct cup-shaped morphology [25] and different sizes (30–300 nm) [26]. On the other hand, the characterization of exosomal protein biomarkers by western blotting is consistent with previous studies claiming that the CD63 level is significantly high in TNBC-derived exosomes [27]. Notably, CD63 is involved in cell survival signaling and the inhibition of apoptosis in TNBC [28].

Moreover, exosome uptake by recipient cells is dependent on their metabolic status, where metabolically active cells such as stem cells may display higher rates of active exosomes uptake than terminally differentiated cells [29]. In this context, the extent of exosome uptake by the cells under investigation revealed that the order of exosomal uptake was MDA-MB-231 cells, followed by HCC1806 cells, and then MCF7 cells. Hence, the strong uptake ability of MDA-MB-231 cells could be attributed to their more mesenchymal-like appearance and greater likelihood of being an invasive subtype of TNBC given their enrichment in epithelial-mesenchymal transition (EMT) and stem-cell markers [30, 31]. This was followed by the uptake ability of HCC1806 cells, which are a more differentiated subtype that has either luminal-like or basal-like morphologies [31, 32]. The last in line is the uptake ability of luminal-A MCF7 cells, where luminal cells are more differentiated and have less ability to migrate due to tight cell-cell junctions [31, 33]. Additionally, TNBC is known to lower the *p*H of its extracellular environment to promote its aggressive features [34, 35]. One of the mechanisms of exosomal uptake is carried out by membrane fusion between exosomes and the plasma membrane of recipient cells, which is an acidic *p*H-dependent process [36, 37]. Hence, exosomal uptake was greater in TNBC cells than in NTNBC cells. Furthermore, it has been demonstrated, as in the present investigations, that TNBC-derived exosomes are more easily taken up by TNBC cells than by NTNBC cells [25, 34]. Thus, it could be concluded that the uptake of exosomes may depend on their origin and receipt.

Morphologically, treatment of TNBC cells with exosomes resulted in the elongation of their spindle-shaped structure. These results are aligned with studies indicating that TNBC-derived exosomes can alter recipient cell morphology to more polarized shapes with more cellular area consistent with motile behavior [39]. However, NTNBC cells showed aggregation after exosome treatment. Previous research showed that TNBC-derived exosomes can decrease cell stiffness and alter cytoskeleton and focal adhesions, thereby changing MCF7 cell morphology [40]. Remarkably, exosome addition to TNBC and NTNBC cells notably enhanced cell proliferation, migration, and colonization. Previous studies have shown that exosomes can form intercellular contacts and develop as multicellular aggregates within recipient cells [41]. Furthermore, TNBC-derived exosomes significantly improve the invasion potential of recipient cells *via* transferring functional cargo molecules. This transfer may facilitate cell proliferation, migration, epithelial-mesenchymal transition (EMT), metastasis, and increase treatment resistance in tumor cells [13, 42].

Morphological changes associated with DOXO treatment markedly affected NTNBC cells compared with TNBC cells. However, cytotoxicity investigations showed that TNBC cells, MDA-MB-231, were more sensitive to DOXO than NTNBC cells, MCF7, as previously reported [43, 44]. Additionally, DOXO induced apoptosis *by* initiating death signaling pathways in susceptible target breast cancer cells [43, 45]. Moreover, DOXO differentially affects breast cancer, TNBC, and NTNBC, where its induction of apoptosis in MCF7 cells [46]. However, whereas examination of MDA-MB-231 cells revealed the presence of rounded, condensed, and detached cells, examination of HCC1806 cells revealed relatively no dead cells. These results align with previous studies reporting that the mesenchymal prototype of TNBC as MDA-MB-231 cells, is more sensitive to DOXO than other prototypes of TNBC, including the basaloid prototype of TNBC, as HCC1806 cells [47]. Consequently, these alterations are consistent with the changes in the tumorigenic behavior of breast cancer cells, as reflected by proliferation, migration, and colonization [43], especially in MDA-MB-231 cells. However, compared to cells treated with DOXO alone, TNBC cells treated with EXO-DOXO exhibited elongated spindle-shaped structures. In the same context, treatment of NTNBC cells with EXO-DOXO slowed the formation of cellular aggregates and clumps. Moreover, treatment of TNBC and NTNBC cells with EXO-DOXO increased the proliferation, migration, and colonization of breast cancer cells. All of these findings may add additional evidence and support for the role of exosomes in the chemoresistance of breast cancer cells to DOXO treatment [48].

In this study, AG490 effectively inhibited the STAT3 pathway across three cell lines, showing the greatest effect in MCF7 cells and the least in MDA-MB-231 cells. The level of inhibition in the breast cancer cell lines was inversely correlated with their sensitivity to AG490. Interestingly, adding EXO to InST-TNBC and InST-NTNBC cells had different effects than using exosomes alone. In InST-MDA-MB231 cells, treatment with EXO decreased the frequency of spindle-shaped cells, while in InST-MCF7 cells, the cell aggregates and clumps disappeared. In contrast, the addition of EXO to InST-HCC1806 cells resulted in a mixture of cells; these cells were composed of small circular cells resembling hematopoietic stem cells, as was the case for increased cell viability and cell death. The proliferation and migration of InST-TNBC and InST-NTNBC cells treated with EXO were less pronounced than those of EXO-treated cells. Concerning colonization, InST-TNBC cells exhibited a reduction in colony formation ability, while in InST-NTNBC cells treated with EXO, colony formation was inhibited. These findings may indicate the enhanced antitumorigenic effects of AG490 through STAT3 inhibition in both subtypes of breast cancer. Moreover, additional evidence may be provided that the tumorigenic influences of exosomes may be mediated by the activity of STAT3 signaling.

Adding DOXO to AG490-treated both subtypes of breast cancer cell lines potentiated the cytotoxic effects of DOXO, causing an increase in the number of dead cells. AG490 was previously demonstrated to be a potential chemotherapeutic agent [49]. The proliferation and migration of AG490-treated breast cancer cells treated with DOXO were less pronounced than those of cells treated with DOXO only. The colony formation ability of InST-TNBC cells was reduced, while that of InST-NTNBC cells was prevented. Thus, consistent with the findings of previous studies [50], the STAT3 pathway inhibition and DOXO treatment may enhance the antitumor effects of these agents in breast cancer cells.

Adding EXO-DOXO to InST-MDA-MB-231 cells did not induce morphological alterations compared to EXO-DOXO treated cells while, compared to InST-DOXO-treated cells, elongated spindle-shaped epithelial cells were observed. In InST-HCC1806 cells, adding EXO-DOXO resulted in more persistent small and circular cells than EXO-DOXO or InST-DOXO treated cells. On the other hand, by adding EXO-DOXO to InST-MCF7 cells, the cellular aggregates and batching of clumps disappeared compared to EXO-DOXO-treated cells. These observations are consistent with the western blotting results showing that AG490 inhibited the pathway, which was more pronounced in NTNBC cells than in TNBC cells. However, compared to InST-DOXO-treated cells, InST-MCF7 cells treated with EXO-DOXO persisted as small circular cells. These observations may further reveal the role of exosomes in the induction of chemoresistance in BC cells to DOXO treatment [48]. Moreover, treatment of InST-TNBC cells with EXO-DOXO caused a decrease in cell proliferation, migration, and colonization compared to EXO-DOXO-treated cells. However, in InST-NTNBC cells, proliferation, and migration were reduced, whereas colonization was completely diminished. These results are in agreement with those of a previous study that demonstrated the role of AG490 in Minimizing the level of phosphorylated “activated” STAT3, leading to a decrease in the ability of these cancer cells to adhere, invade, and metastasize [51]. On the other hand, concerning proliferation ability, EXO-DOXO treatment of both InST-TNBC and InST-NTNBC cells, compared to InST-DOXO-treated cells, had various effects. For instance, treatment of InST-MDA-MB-231 cells with EXO-DOXO slightly increased cell proliferation. In contrast, treatment with InST-HCC1806 decreased cell proliferation. Treatment of InST-MCF7 cells did not change cell proliferation. However, the cellular migration of InST-TNBC and InST-NTNBC cells treated with EXO-DOXO was greater than that of InST-DOXO-treated cells. Compared to InST-DOXO-treated cells, colony formation was more efficient in InST-MDA-MB-231 cells after EXO-DOXO treatment, while only slight colony formation was observed in InST-HCC1806 cells. Conversely, treatment of InST-MCF7 cells did not change the colony formation capacity. This, in turn, may further support the suggestion that exosomes may have the capacity to transfer and maintain the stem cell phenotype in recipient cells [52, 53]. Hence, cancer stem cells can cease proliferating and survive in a quiescent state. Consequently, these cells may wait for appropriate environmental conditions to proliferate again, giving rise to metastasis [54].

In conclusion, all of these observations are aligned with those of previous studies and reflect the influence of exosomes on the enhancement of tumorigenic behavior and chemotherapeutic resistance in breast cancer cells, especially in TNBC cells [55, 56].

In the molecular biochemistry of cancer, regarding Wnt/β-catenin signaling, it has been demonstrated the tumorigenic role of the β-catenin molecule in breast cancer, as elevated levels of nuclear and/or cytoplasmic β-catenin are detected in breast tumor tissues [57]. Moreover, elevated levels of β-catenin and its translocation to the nucleus, where it interacts with TCF/LEF cotranscription factors, activate β-catenin target genes implicated in cancer cell proliferation, EMT, migration, and angiogenesis [58, 59]. For instance, Cyclin D1 is a crucial regulator of the G1-S transition checkpoint of the cell cycle, and its abnormal upregulation is implicated in carcinogenesis and progression of human breast carcinoma [20]. Furthermore, β-catenin induces the transcription of the Axin2 gene, which is known to be a negative regulator of the signaling pathway, where it stimulates the phosphorylation and degradation of β-catenin, limiting the duration or intensity of WNT-initiated signaling. Thus, it plays a complementary role by being transcribed following the activation of the WNT/β-catenin signaling pathway and subsequently creating a negative feedback loop to silence the signaling pathway following transduction of the WNT signal [60, 61].

Previously, it was mentioned that exosomal packaging and delivery of proteins can regulate different cellular signaling pathways [62]. Thus, the observed modulations in the WNT signaling pathway in TNBC and NTNBC cells under the influence of EXO treatment agree with this concept. EXO treatment of MDA-MB-231 cells significantly increased β-catenin, Cyclin D1, WNT1, and AXIN2 expression, while, in HCC1806 cells increased only β-catenin expression, moreover, in MCF7 cells, significantly increased β-catenin and Cyclin D1 expression. Thus, treatment with EXO activates the WNT signaling pathway, which may provoke tumorigenic behavior in these cells. However, the modulatory effect of EXO on the WNT signaling pathway varies from one cell type to another, although the number of applied exosomes is fixed. In this respect, several factors may be involved, including the nature of the recipient cells and the degree of exosomal uptake, e.g., the putative specificity of exosomes for certain cell types and the complexity of the function of exosomes in intercellular communication [63] (see above).

The involvement of the WNT signaling pathway in TNBC chemoresistance has been reported [64, 65]. In the present study, DOXO treatment of MDA-MB-231 cells significantly upregulated the expression of β-catenin, Cyclin D1, and AXIN2, which was associated with the downregulation of WNT1 expression. However, treatment with DOXO resulted in a significant downregulation of WNT1 and upregulation of Cyclin D1 expression in HCC1806 cells, whereas in MCF7 cells, a significant upregulation of Cyclin D1 expression was observed. The influence of DOXO on the different cells utilized in the present study can be seen in the view that although WNT1 proves the capacity of the WNT/β-catenin pathway to initiate breast cancer, the WNT1 protein is hardly overexpressed in human breast cancer [66]. Additionally, other studies have reported that the proportion of tumors in which a given WNT gene is overexpressed is generally low [67]. The upregulation of Cyclin D1 expression in HCC1806 and MCF7 cells despite the unchanged β-catenin expression may be attributed to the impact of signaling pathways other than the WNT signaling pathway in those cells [20].

Interactions between the components of exosomes and the active metabolites of DOXO may positively or negatively affect the WNT signaling pathway. Thus, compared to DOXO-treated cells, MDA-MB-231 cells treated with EXO-DOXO exhibited significantly decreased β-catenin, Cyclin D1, and AXIN2 and increased WNT1 expressions. Moreover, treatment of HCC1806 and MCF7 cells with EXO-DOXO significantly upregulated β-catenin and WNT1 expression. No significant change in AXIN2 expression was observed in HCC1806 cells, while in MCF7 cells, the AXIN2 expression was significantly lower than in DOXO-treated cells. These findings may provide additional insight into the crucial role of exosomes in chemoresistance and other tumorigenic aggressive behaviors. Previously, it was suggested that exosome expulsion of anticancer agents may provide another barrier to the proper action of most therapeutic agents or their intracellular metabolites, leading to reduced efficacy [68]. However, further studies are needed to clarify the impact of EXO on the chemotherapeutic efficacy of or chemoresistance to DOXO.

Functional interactions between the WNT and STAT3 pathways in tumor initiation and metastasis have been reported [69, 70]. However, it remains unclear whether these pathways operate within the same or different cells in tumors and how they coordinate at the molecular level during progression [71]. It has been suggested that interactions may be cell type-specific [72]; for example, in esophageal squamous cell carcinoma, STAT3 is influenced by the β-catenin/T-cell factor pathway [73], while in breast cancer, STAT3 may directly regulate β-catenin transcription, demonstrating a potential synergy in their oncogenic roles [74]. Thus, given that both STAT3 and β-catenin are activated in a subset of breast tumors, it was hypothesized that STAT3 may provide a further mechanism by which β-catenin is regulated in breast cancer cells [74]. Notably, the results and observations of the present molecular investigations are consistent with this assumption.

Thus, treatment of InST-MDA-MB-231 cells with EXO significantly reduced the β-catenin, Cyclin D1, WNT1, and AXIN2 expression compared to EXO-treated cells. Moreover, in InST-HCC1806 cells, significantly decreased β-catenin expression only. However, in InST-MCF7 cells, increased WNT1 levels compared to EXO-treated cells. Moreover, in InST-MDA-MB-231 cells, DOXO treatment significantly reduced the β-catenin, CyclinD1, WNT1, and AXIN2 expression, however in InST-HCC1806 cells, increased WNT1 expression only compared to DOXO-treated cells. Interestingly, in InST-MCF7 cells, treatment with DOXOs significantly increased the β-catenin, WNT1, and AXIN2 expression; in contrast, the cyclin D1 expression was reduced. Furthermore, adding EXO-DOXO to InST-MDA-MB-231 cells significantly reduced the β-catenin, WNT1, and AXIN2 expression while, in InST-HCC1806 cells, decreased β-catenin and AXIN2 expression compared to EXO-DOXO treated cells. In InST-MCF7 cells, WNT1 expression was decreased, whereas AXIN2 expression was increased. However, compared to those in InST-DOXO-treated cells, the β-catenin and AXIN2 expression in InST-MDA-MB-231 cells treated with EXO-DOXO were significantly lower; on the other hand, the expression level of WNT1 was increased. Moreover, in InST-HCC1806 cells treated with EXO-DOXO, the AXIN2 expression level was significantly decreased. However, in InST-MCF7 cells, treatment with EXO-DOXO significantly decreased β-catenin, WNT1, and AXIN2 expression.

In addition, crosstalk between exosomes and the Notch signaling pathway was described by assessing the targeted genes NOTCH1, NOTCH2, JAG1, HES1, and DLL4. Biochemical analysis of MDA-MB-231 cells revealed that EXO treatment significantly upregulated NOTCH2, JAG1, HES1, and DLL4 expression, while compared with no treatment, treatment with EXO significantly upregulated NOTCH1, NOTCH2, and HES1 expression. However, treatment of MCF7 cells with EXO did not significantly change the expression of the studied Notch pathway signaling genes. Notch receptor expression and activation are linked to aggressive breast cancer features, like invasion and chemoresistance, especially in TNBC [23]. Other studies connect Notch signaling to TNBCs, particularly the basal-like and mesenchymal stem-like subtypes, which show high receptor levels and poor tumor outcomes [75–78]. Thus, considering the role of exosomes in promoting tumorigenic behavior and the vital role of the Notch signaling pathway in promoting tumor progression, it could be concluded that exosomes enhance tumorigenic behavior in TNBC, partly through the Notch signaling pathway. Concerning NTNBC, the tumorigenic behavior could occur through signaling pathways other than the Notch pathway. It could be attributed to either the clinical characteristics of the recipient cells or the biological features of the cells of origin.

Regarding DOXO treatment, the treatment of MDA-MB-231 cells significantly upregulated NOTCH2, JAG1, HES1, and DLL4 expression compared to untreated cells, moreover in HCC1806 cells upregulated NOTCH1 but downregulated JAG1 expression. Previously, treatment with DOXO was shown to intensely increase the expression of multiple NOTCH pathways in cancer cells [79, 80]. Moreover, aberrant NOTCH signaling has also been extensively linked to TNBC since Notch subtype receptor overexpression is related to the aggressive, metastatic, and therapeutic resistance phenotypes of TNBC [23, 81]. Notably, JAG1 has been reported to signal to tumor cells [82, 83] to promote angiogenesis and tumor growth via the mitogen-activated protein kinase (MAPK) pathway [84, 85]. Moreover, DOXO has been demonstrated to exert its antitumor effects by inducing apoptosis and inhibiting angiogenesis [86], possibly through the downregulation of JAG1 expression. On the other hand, treatment of MCF7 cells with DOXO significantly downregulated the expression of HES1 and DLL4. Thus, as previously demonstrated, high doses of DOXO (≥ 0.5 µM) may inhibit the Notch signaling pathway [79, 87], as observed in MCF7 cells.

However, interestingly, the results of the present study may indicate that the interaction between EXOs and DOXO may be influenced by the pathological characteristics of the BC recipient cells. Thus, compared to DOXO-treated cells, MDA-MB-231 cells treated with EXO-DOXO exhibited significant decreases in JAG1, HES1, and DLL4 expression, while in HCC1806 cells, treatment significantly upregulated NOTCH2 and JAG1 expressions. However, in MCF7 cells, EXO-DOXO treatment significantly upregulated NOTCH1, NOTCH2, and JAG1 expressions. Therefore, treatment of MDA-MB-231 cells with EXO-DOXO may provoke the antitumor effects of DOXO. However, treatment of either HCC1806 or MCF7 cells with EXO-DOXO may enhance the tumorigenic behavior of BC cells, especially resistance to therapy. In this regard, exosomes may induce resistance to chemotherapy through direct drug export, trafficking of drug efflux pumps, and cell-to-cell exchange of miRNAs [88, 89]. Accordingly, further studies are needed to confirm and clarify the mechanisms behind these observations.

Additionally, crosstalk between the STAT and Notch signaling pathways has been described and shown to have pleiotropic impacts on many popular processes regulating cell fate [82, 90]. Thus, in the present study, in InST-TNBC cells, EXO treatment significantly downregulated the expression of all studied genes in the Notch signaling pathway. In contrast, InST-NTNBC upregulated the expression of all studied genes in the Notch signaling pathway compared to that in cells treated with EXO only without STAT3 inhibition.

Moreover, in InST cells, treatment of MDA-MB-231 cells with DOXO significantly downregulated the expression of all studied genes involved in Notch signaling compared, while in InST-HCC1806 cells downregulated NOTCH2 expression only. In contrast, in InST-MCF7 cells, DOXO upregulated the expression of all the studied genes in the Notch signaling pathway compared to DOXO-treated cells without inhibition. Furthermore, upon comparing the gene expression of InST-cells treated with EXO-DOXO to cells treated with EXO-DOXO, the results revealed that, in InST-MDA-MB-231 cells, all the studied genes were significantly downregulated. Additionally, in InST-HCC1806 cells, significant decreases in the NOTCH1, NOTCH2, and JAG1 expression were detected. In InST-MCF7 cells, NOTCH1 and DLL4 expression was significantly upregulated compared to that in EXO-DOXO-treated cells. Additionally, compared to InST-DOXO cells, InST-MDA-MB-231 cells treated with EXO-DOXO exhibited significantly downregulated NOTCH1, NOTCH2, JAG1, and DLL4 expression. Moreover, in InST-HCC1806 cells, only NOTCH1 expression was significantly downregulated. However, in InST-MCF7 cells, EXO-DOXO treatment significantly upregulated NOTCH1 and DLL4 expression. Taken together, these findings may indicate that in TNBC, exosomes may exert their tumorigenic effects through the Notch signaling pathway mediated by the STAT3 pathway. Moreover, in NTNBC, EXO may exert its tumorigenic effects through the Notch signaling pathway independently of the STAT3 pathway. These findings may be supported by Piwarski et al., who reported the failure of STAT3 Phosphorylation in NTNBC cells and MCF7 cells [91].

## Conclusion

The present study is an effort to determine the role of exosomes in enhancing and potentiating the tumorigenic behavior of TNBC. Additionally, a study was executed to determine the mechanism(s) through which exosomes play a tumorigenic role in TNBC. In this respect, the role of the WNT and Notch signaling pathways mediated by the STAT3 pathway was examined. The present investigation may provide additional evidence and support for the crucial role of exosomes in promoting aggressive TNBC behaviour. In addition, these results may indicate the role of the WNT signaling pathway, which is mediated by STAT3, as one of the mechanisms involved in exhibiting tumorigenic aggressive characteristics influenced by exosomes in both TNBC and NTNBC cells. On the other hand, exosomes may exert their tumorigenic effects through the Notch signaling pathway mediated by the STAT3 pathway in TNBC. Moreover, in NTNBC, exosomes may exert their tumorigenic effects through the Notch signaling pathway independently of the STAT3 pathway.

## Supplementary Information

Below is the link to the electronic supplementary material.

Supplementary Material 1

## Data Availability

Data available on request from the corresponding author.

## Abbreviations

Alix: ALG-2-interacting protein-X
ATG16L: Autophagy related 16 like 1
ATG5: Autophagy related 5
CD: Cluster of differentiation
DMEM: Dulbecco’s modified Eagle’s medium
DMSO: Dimethyl sulfoxide
DOXO: Doxorubicin
ELISA: Enzyme-linked immunosorbent assay
ER: Estrogen receptor
EXO: Exosomes
FBS: Fetal bovine serum
Her2: Protein-human epidermal growth factor receptor 2
InST cells: Inhibited STAT3 signaling cells
MTT: 3-[4,5-dimethylthiazol-2-yl]-2,5 diphenyl tetrazolium bromide
Non-TNBC: Non- triple negative breast cancer
NOTCH: Neurogenic locus notch homolog protein
PI: Propidium iodide
PR: Progesterone receptor
qRT: PCR-Quantitative real time polymerase chain reaction
STAT3: Signal transducer and activator of transcription 3
TNBC: Triple negative breast cancer
TSG101: Tumor susceptibility gene-101
Un-InST cells: Uninhabited STAT3 signaling cells
WNT: Wingless-related integration site
