# Supplementary material for: The involvement of exosomes in the tumorigenicity of breast cancer cell lines through the crosstalk between STAT3, Notch, and Wnt signaling pathways
Source: Discov Oncol. 2025 Sep 15;16:1685. doi: 10.1007/s12672-025-03334-0 (PMC12436252; doi:10.1007/s12672-025-03334-0)

Supplementary Figure 1. Representative original image of western blot for exosomal surface marker protein detection Lanes (1-6): exosomes isolated from serum TNBC sample. The imaged were scanned using a Gel DoxXR+Gel Documentation System (Bio-Rad). CD63 protein was highly expressed compared to the PCD6IP (Alix) and TSG101 proteins. **Color-coded prestained Protein Marker, Broad Range (10-250 kDa, #74124 was used.**

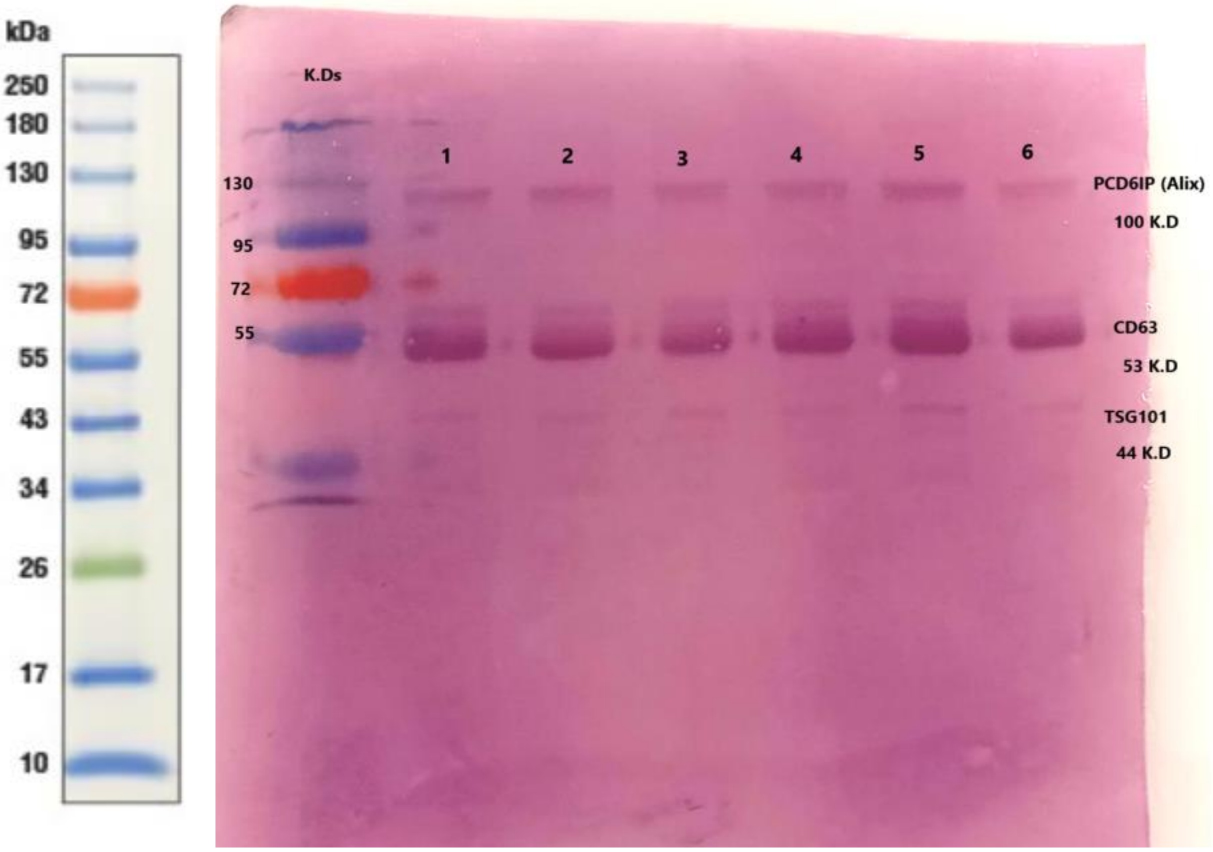

Supplementary Figure 2A. Western blotting results. (a) Representative original image of western blot for MDA-MB-231 and HCC 180 cells before and after STAT3 pathway inhibition, by AG490, scanned by Gel Dox XR+ Gel Documentation System (BIO-RAD Laboratories, Inc). (b-j) Chromatogram of lanes; the levels Pan-STAT3 and phospho-STAT were measured using the area under each peak.

MDA-MB-231 and HCC1806 cells

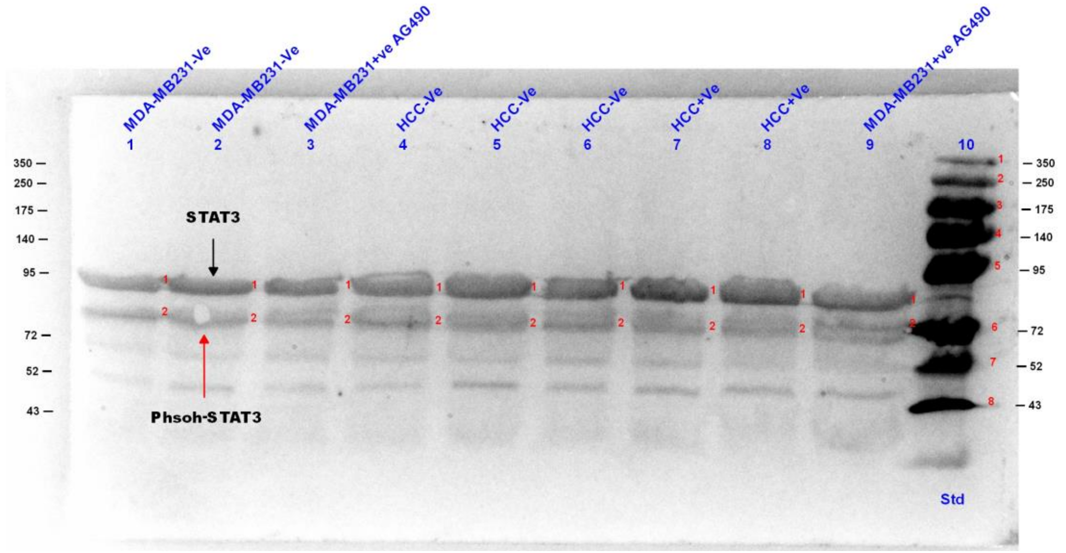

(a)

Lane 1 - MDA-MB231-Ve

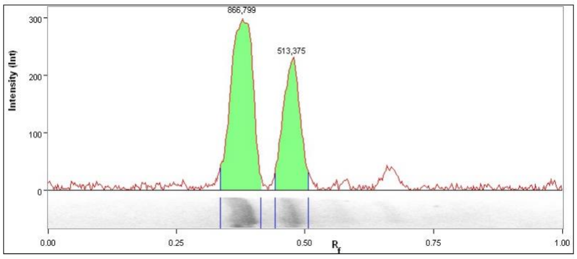

Band No.	Band Label	Mol. Wt. (KDa)	Relative Front	Volume (Int)	Abs. Quant.	Rel. Quant.
1		90.4	0.380	866,799	N/A	N/A
2		78.4	0.476	513,375	N/A	N/A

(b)

Lane 2 - MDA-MB231-Ve

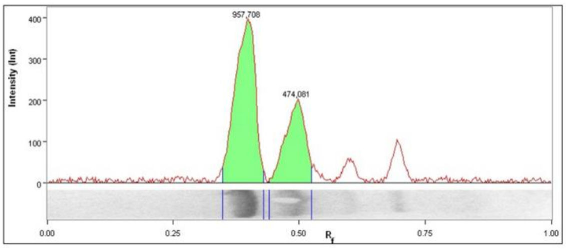

Band No.	Band Label	Mol. Wt. (KDa)	Relative Front	Volume (Int)	Abs. Quant.	Rel. Quant.
1		88.1	0.397	957,708	N/A	N/A
2		76.1	0.496	474,081	N/A	N/A

(c)

Lane 3 - MDA-MB231+ve AG490

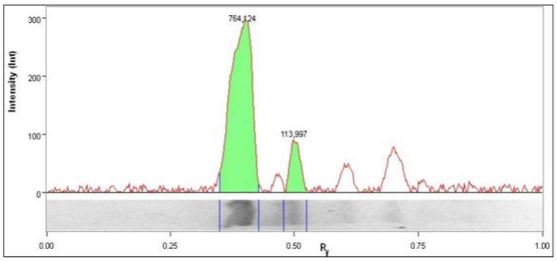

Band No.	Band Label	Mol. Wt. (KDa)	Relative Front	Volume (Int)	Abs. Quant.	Rel. Quant.
1		88.0	0.398	764,124	N/A	N/A
2		75.4	0.502	113,997	N/A	N/A

(d)

Lane 9 - MDA-MB231+ve AG490

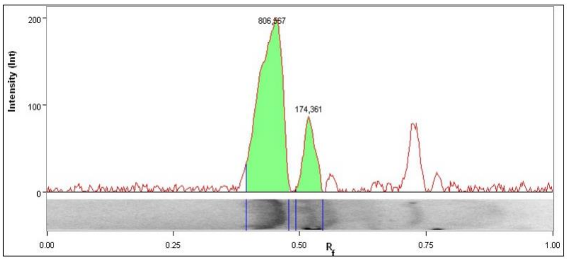

Band No.	Band Label	Mol. Wt. (KDa)	Relative Front	Volume (Int)	Abs. Quant.	Rel. Quant.
1		81.7	0.448	806,567	N/A	N/A
2		73.4	0.520	174,361	N/A	N/A

(e)

Lane 4 - HCC-Ve

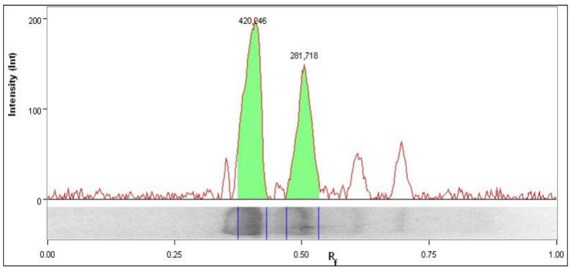

Band No.	Band Label	Mol. Wt. (KDa)	Relative Front	Volume (Int)	Abs. Quant.	Rel. Quant.
1		86.9	0.406	420,246	N/A	N/A
2		75.0	0.506	281,718	N/A	N/A

(f)

Lane 5 - HCC-Ve

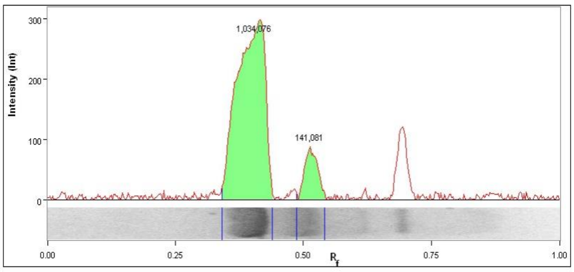

Band No.	Band Label	Mol. Wt. (KDa)	Relative Front	Volume (Int)	Abs. Quant.	Rel. Quant.
1		87.1	0.405	1,034,076	N/A	N/A
2		74.1	0.514	141,081	N/A	N/A

(g)

Lane 6 - HCC-Ve

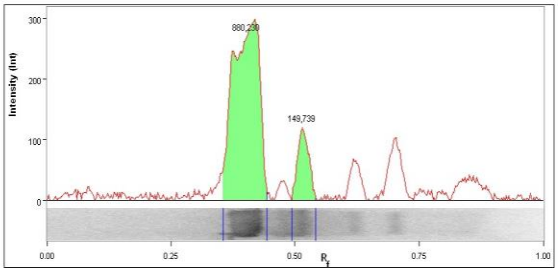

Band No.	Band Label	Mol. Wt. (KDa)	Relative Front	Volume (Int)	Abs. Quant.	Rel. Quant.
1		87.3	0.403	880,230	N/A	N/A
2		74.0	0.516	149,739	N/A	N/A

(h)

Lane 7 - HCC+Ve

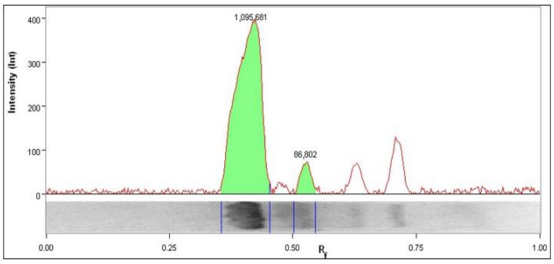

Band No.	Band Label	Mol. Wt. (KDa)	Relative Front	Volume (Int)	Abs. Quant.	Rel. Quant.
1		85.5	0.418	1,095,681	N/A	N/A
2		72.7	0.527	86,802	N/A	N/A

(i)

Lane 8 - HCC+Ve

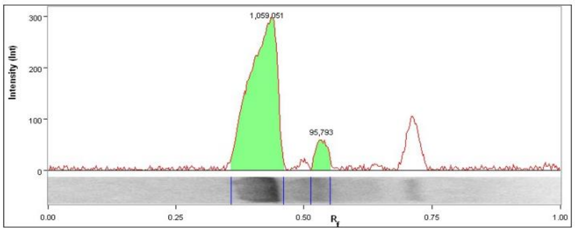

Band No.	Band Label	Mol. Wt. (KDa)	Relative Front	Volume (Int)	Abs. Quant.	Rel. Quant.
1		83.8	0.431	1,059,051	N/A	N/A
2		71.5	0.536	95,793	N/A	N/A

(j)

Supplementary Figure 2B. Western blotting results. (a) Representative original image of western blot for MCF7 cells before and after STAT3 pathway inhibition, by AG490, scanned by Gel Dox XR+ Gel Documentation System (BIO-RAD Laboratories, Inc). (b-g) Chromatogram of lanes; the levels Pan-STAT3 and phospho-STAT were measured using the area under each peak.

MCF7 cells

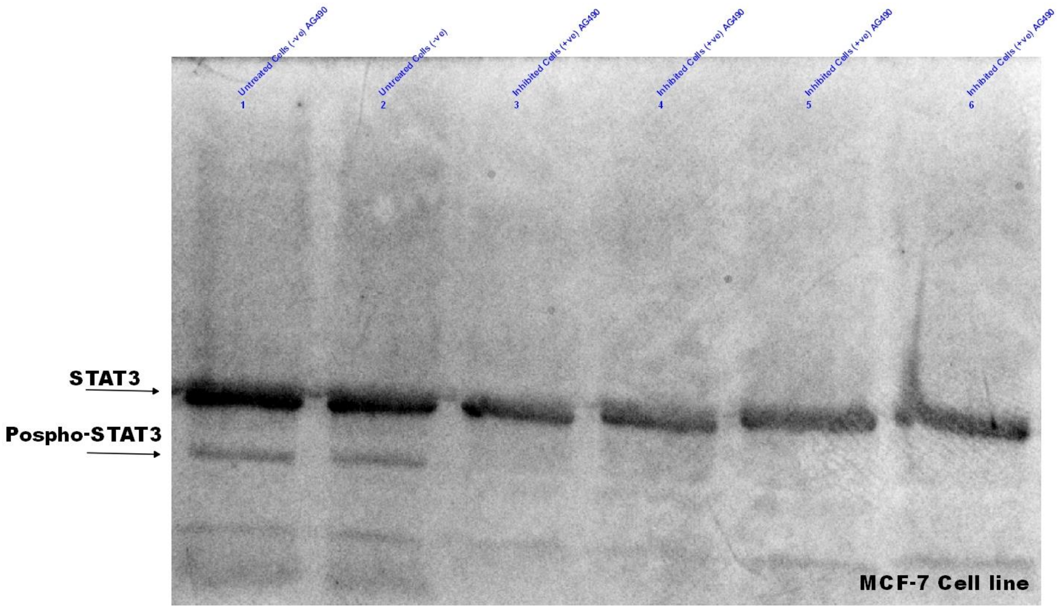

(a)

Lane 1 - Untreated Cells (-ve) AG490

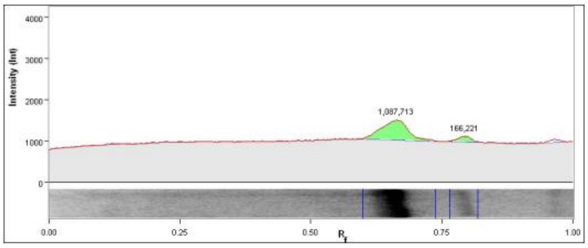

Band No.	Band Label	Mol. Wt. (KDa)	Relative Front	Volume (Int)	Abs. Quant.	Rel. Quant.
1		N/A	0.664	1,087,713	N/A	N/A
2		N/A	0.794	166,221	N/A	N/A

(b)

Lane 2 - Untreated Cells (-ve)

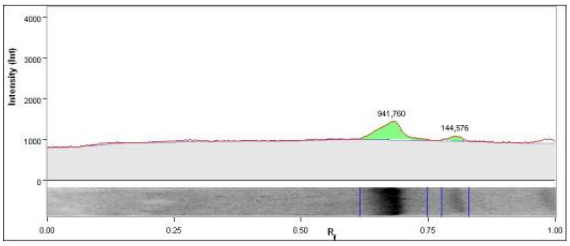

Band No.	Band Label	Mol. Wt. (KDa)	Relative Front	Volume (Int)	Abs. Quant.	Rel. Quant.
1		N/A	0.681	941,760	N/A	N/A
2		N/A	0.806	144,576	N/A	N/A

(c)

Lane 3 - Inhibited Cells (+ve) AG490

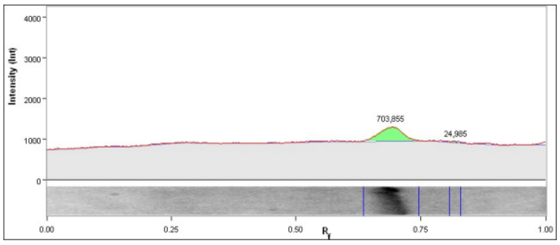

Band No.	Band Label	Mol. Wt. (KDa)	Relative Front	Volume (Int)	Abs. Quant.	Rel. Quant.
1		N/A	0.692	703,855	N/A	N/A
2		N/A	0.822	24,985	N/A	N/A

(d)

Lane 4 - Inhibited Cells (+ve) AG490

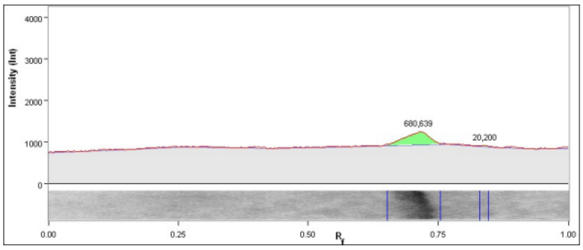

Band No.	Band Label	Mol. Wt. (KDa)	Relative Front	Volume (Int)	Abs. Quant.	Rel. Quant.
1		N/A	0.714	680,639	N/A	N/A
2		N/A	0.842	20,200	N/A	N/A

(e)

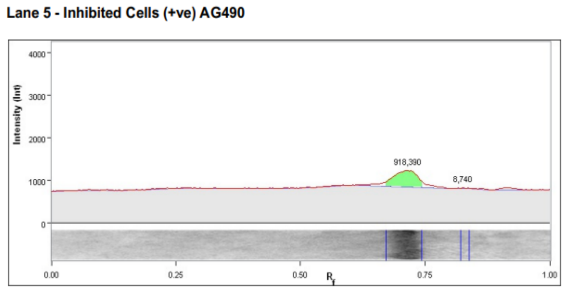

Band No.	Band Label	Mol. Wt. (KDa)	Relative Front	Volume (Int)	Abs. Quant.	Rel. Quant.
1		N/A	0.717	918,390	N/A	N/A
2		N/A	0.828	8,740	N/A	N/A

(f)

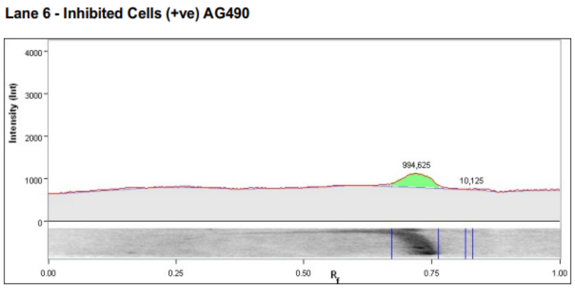

Band No.	Band Label	Mol. Wt. (KDa)	Relative Front	Volume (Int)	Abs. Quant.	Rel. Quant.
1		N/A	0.722	994,625	N/A	N/A
2		N/A	0.833	10,125	N/A	N/A

(g)

Supplementary Figure 3. Representative original image of western blot for GAPDH (Novus Biologicals, LLC, Ca.no **NB100-56875**) as a reference protein. Lanes. The imaged were scanned using a Gel DoxXR+Gel Documentation System (Bio-Rad). Color-coded prestained Protein Marker, Broad Range (10-250 kDa, #74124 was used.

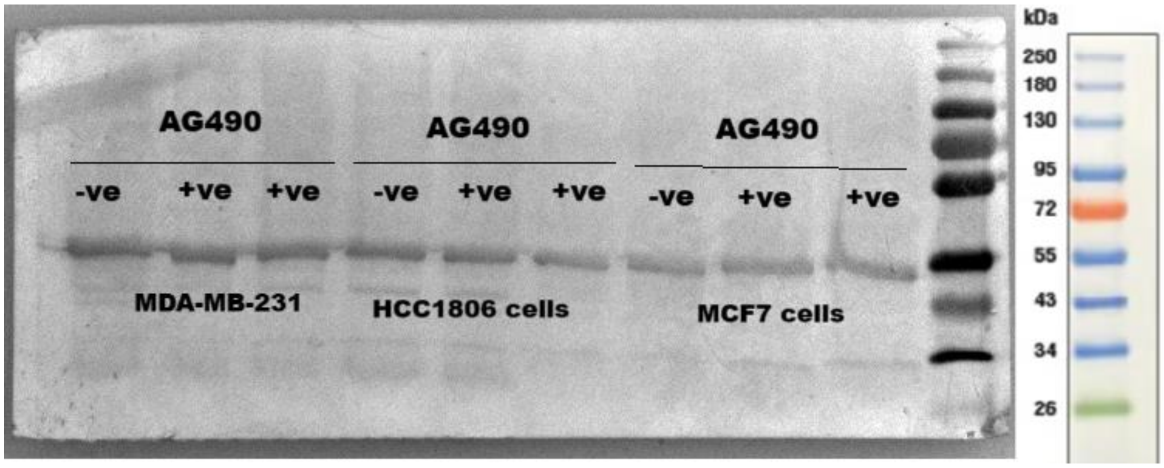

Supplement: Supplementary file 1 — Supplementary Material 1 [file 12672_2025_3334_MOESM1_ESM.pdf]
